# InMAP: A model for air pollution interventions

**DOI:** 10.1371/journal.pone.0176131

**Published:** 2017-04-19

**Authors:** Christopher W. Tessum, Jason D. Hill, Julian D. Marshall

**Affiliations:** 1 Department of Civil and Environmental Engineering, University of Washington, Seattle, Washington, United States of America; 2 Department of Bioproducts and Biosystems Engineering, University of Minnesota, St. Paul, Minnesota, United States of America; Universidade de Vigo, SPAIN

## Abstract

Mechanistic air pollution modeling is essential in air quality management, yet the extensive expertise and computational resources required to run most models prevent their use in many situations where their results would be useful. Here, we present InMAP (Intervention Model for Air Pollution), which offers an alternative to comprehensive air quality models for estimating the air pollution health impacts of emission reductions and other potential interventions. InMAP estimates annual-average changes in primary and secondary fine particle (PM_2.5_) concentrations—the air pollution outcome generally causing the largest monetized health damages–attributable to annual changes in precursor emissions. InMAP leverages pre-processed physical and chemical information from the output of a state-of-the-science chemical transport model and a variable spatial resolution computational grid to perform simulations that are several orders of magnitude less computationally intensive than comprehensive model simulations. In comparisons run here, InMAP recreates comprehensive model predictions of changes in total PM_2.5_ concentrations with population-weighted mean fractional bias (MFB) of −17% and population-weighted *R*^2^ = 0.90. Although InMAP is not specifically designed to reproduce total observed concentrations, it is able to do so within published air quality model performance criteria for total PM_2.5_. Potential uses of InMAP include studying exposure, health, and environmental justice impacts of potential shifts in emissions for annual-average PM_2.5_. InMAP can be trained to run for any spatial and temporal domain given the availability of appropriate simulation output from a comprehensive model. The InMAP model source code and input data are freely available online under an open-source license.

## Introduction

Ambient air pollution is estimated to kill over three million people per year globally [1, 2]. Reducing air pollution and its impacts is therefore an important policy goal. However, it is often unclear a priori which potential emission reductions would be most effective in improving air pollution and health because the chemical and physical relationships between emissions of air pollutants and the ambient concentrations that result can be complex [3]. To assist in decision-making, air pollution models are often used to estimate the health effects of a range of hypothetical changes in emissions.

Eulerian Chemical Transportation Models (CTMs; examples: CAMx, [4]; CMAQ, [5]; WRF-Chem, [6]; GATOR-GCMOM, [7]) are powerful tools that can simulate the effectiveness of emission reductions at reducing air quality-related health impacts. Running CTM simulations generally requires dedicated experts or teams, and often is computationally expensive and time consuming. For example, a single simulation for annual exposure in the contiguous US with a 12 km spatial resolution can take days to run on a high performance computing system (i.e., a “super-computer”) [8].

The computational intensity and high degree of difficulty inherent in performing CTM simulations is a bottleneck for the rate at which air quality strategies can be evaluated, for the number of people who can perform such evaluations, and also therefore potentially the rate at which policies for improving air quality can be investigated, evaluated, potentially enacted. Therefore, there is a need for air quality models that are simpler to use; provide results more quickly than CTMs, while minimizing losses in predictive accuracy; and potentially can be run by outside experts. Here, we describe such a model.

The design of our new model reflects current understandings of the health impacts of air pollution:

1. Of the three million global deaths per year attributed to ambient air pollution, approximately 95% are caused by fine particulate matter (PM_2.5_) [1, 2]. The strongest predictor for these deaths is chronic PM_2.5_ exposure over periods of a year or more [9–11]. Therefore, a prediction of chronic exposure to PM_2.5_ is a good indicator of overall health impacts from air pollution.
2. PM_2.5_ can travel long (e.g., intercontinental) distances but can also be highly spatially variable near emissions sources. Additionally, PM_2.5_ can be both directly emitted (“primary”) and formed in the atmosphere (“secondary”). Models that predict PM_2.5_ exposure should consider all of these aspects.
3. Air pollution-mediated health damages can be a major driver of overall environmental externalities [12, 13]. Therefore, air pollution models that can be used by non-air-pollution-experts can be beneficial.

Numerous air quality models already exist that have lower operational difficulty than CTMs [14–30]. As discussed in S1 Appendix, while each model type is well-suited to certain use-cases, none are ideal for the specific use-case we are interested in: an adaptable and updatable model for human health impacts of changes in air pollutant emissions that can resolve intraurban gradients in pollution concentrations near emissions sources, can track the long-range transport of pollution, and can be used by non-specialists.

Here we develop and apply a new approach, which we implement as the Intervention Model for Air Pollution (InMAP). InMAP is designed to provide estimates of air pollution health impacts resulting from marginal changes in pollutant emissions, such as those resulting from new regulations. InMAP combines spatially-resolved annual-average physical and chemical information derived from a state-of-the-science CTM (WRF-Chem) with simplifying assumptions regarding atmospheric chemistry for cases of marginal changes in emissions. InMAP is developed here to predict changes in annual average exposure to PM_2.5_; as mentioned above, that outcome is estimated to cause 95% of air quality-related mortalities. The model is also able to predict changes in concentrations of several other pollutants. Features of InMAP include reductions in computational cost relative to CTMs, yet with more spatially detailed results than are available with existing reduced-complexity models, a variable-resolution grid that focuses on human exposures by employing higher spatial resolution in urban areas and lower spatial resolution in rural and remote locations and at high altitude; and the ability to account for spatially variable aspects of secondary PM_2.5_ formation while also being amenable to running many scenarios and theoretically simple enough for use by non-experts. InMAP is designed to be informed by the default output of a single CTM run, so CTM runs that were originally created for other purposes can be used to create InMAP inputs. To our knowledge, the modeling approach developed here is the first of its kind for air pollution. It was inspired by recent advancements in reduced complexity sediment transport modeling [31, 32].

## Methods

### Model formulation

The fate and transport of pollution in the atmosphere can be represented by a reaction-advection-diffusion equation:

$$\frac{\partial C_{i}}{\partial t}=\nabla \cdot \left(D\nabla C_{i}\right)-\nabla \cdot \left(\vec{v}C_{i}\right)+\sum_{j=1}^{n}R_{i,j}+E_{i}-d_{i}$$ (1)

where *C*_*i*_ is the concentration of one of *n* model pollutant species, *D* is a molecular diffusion coefficient (neglected here as a negligible source of chemical transport in the atmosphere compared to advection), $\vec{v}$ is the wind vector, $\sum_{j=1}^{n}R_{i,j}$ is the net formation rate of species *i* from species *j*, *E*_*i*_ is pollutant emission, and *d*_*i*_ is pollutant removal via wet and dry deposition. InMAP estimates pollutant concentrations by estimating a steady-state solution to Eq (1), yielding annual average pollutant concentration results. To do so, we replace each of the terms on the right-hand side of Eq (1) with parameterizations suitable for numerical solution as described below.

InMAP solves Eq (1) for model chemical species comprised of primary PM_2.5_, volatile organic compounds (VOCs), secondary organic aerosol (SOA), sulfur dioxide (SO_*x*_), particulate sulfate (*p*SO_4_), oxides of nitrogen (NO_*x*_), particulate nitrate (*p*NO_3_), ammonia (NH_3_), and particulate ammonium (*p*NH_4_). InMAP assumes that primary PM_2.5_, VOCs, SOA, and SO_*x*_, NO_*x*_, and NH_3_ can be emitted directly; the other species are secondary products formed in the atmosphere. InMAP assumes atmospheric particle diameter and density—which it only uses to calculate dry deposition rate—to be constant at 0.3 *μ*m and 1830 kg m^−3^, respectively.

#### Spatial discretization

**Fig 1:**
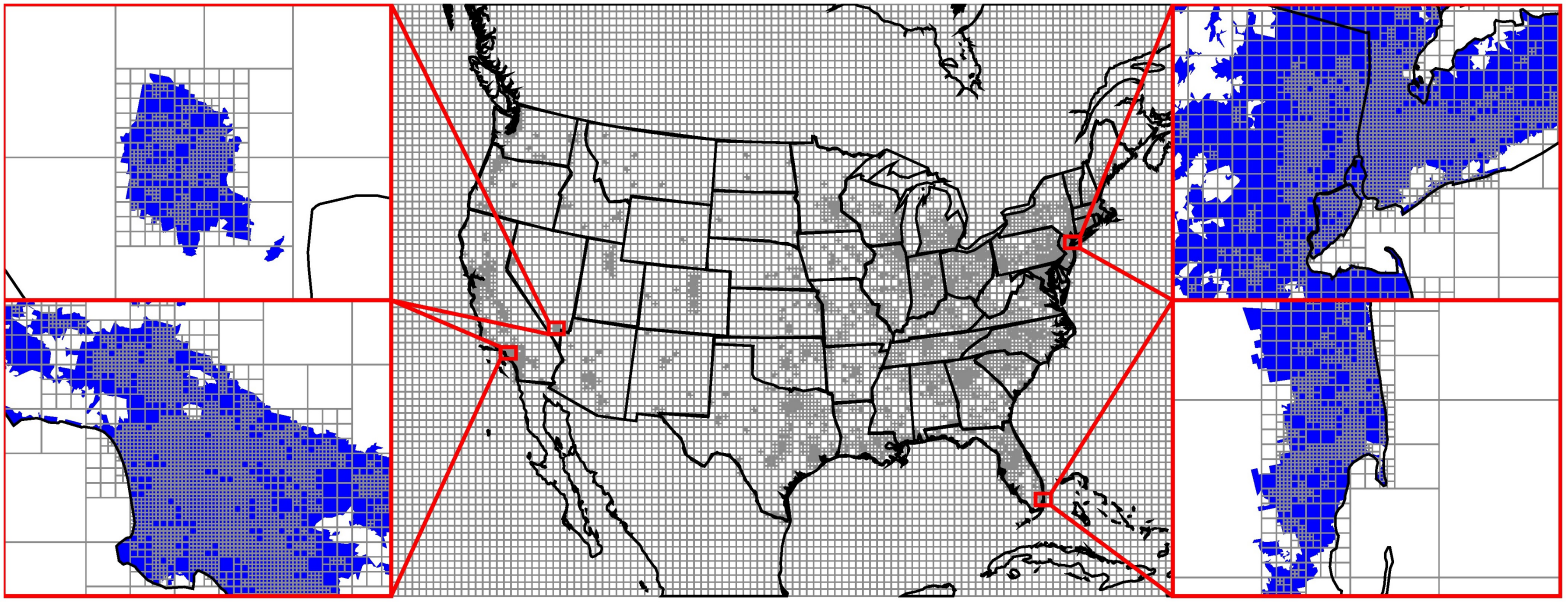
Spatial discretization of the model domain into variable resolution grid cells. The insets show the areas around the cities of Las Vegas, Los Angeles, New York, and Miami in detail. Blue shading represents urban areas as defined by the US Census.

Air pollution model simulations with increased spatial resolution can potentially provide improved exposure predictions [33] and often yield higher overall health impact estimates [34, 35]. CTMs typically employ a regular (i.e., constant-resolution) horizontal grid; to increase spatial resolution over important areas they may use a small number of higher-resolution “nested” grids inside a lower resolution outer grid. InMAP instead employs a variable resolution rectangular grid where grid cell size varies throughout the domain. To focus computational resources on understanding exposures and health impacts, InMAP users can choose one of two grid cell size strategies. With the first option, grid cells are smaller in urban areas and larger in rural and remote areas. Horizontal resolution also varies with height: because horizontal variability in concentrations decreases with height above the ground, we employ a low-resolution horizontal grid for all cells above a specific height (here, set to approximately 1500 m). With the second option, grid cell size varies dynamically while the simulation is running based on gradients in population density and pollutant concentration. Fig 1 shows a result of applying the first option algorithm for grid cell sizes. In our simulations, we use a spatial domain that covers the contiguous US, southern Canada, and northern Mexico, with grid cell edge lengths ranging between 1 and 48 km. The results presented here use the second grid cell size algorithm as it tends to give shorter model run times, but both options yield very similar results. Both algorithms and sample run times are described in detail in S2 Appendix.

#### Temporal discretization

Instead of solving for pollutant concentrations at specific points in time using temporally explicit input data as CTMs do, InMAP directly estimates annual average pollutant concentrations using annual average input data and numerical integration. We selected this approach because, as mentioned above, the vast majority of monetized damages from air pollution are attributable to human mortality from chronic (annual or longer) exposure to PM_2.5_.

To reach a steady-state solution, InMAP starts with an initial estimate of the changes in concentrations caused by an emissions scenario (the initial estimate is that there are no changes in concentrations) and iterates the model forward in time until the concentrations converge to a steady-state solution (i.e., until the predicted concentrations no longer change as the model continues to run). The integration time step Δ*t* is chosen using the Courant–Friedrichs–Lewy condition [36] as in Eq (2):

$$\Delta t=\frac{C_{\text{max}}}{\sqrt{3}}\;{\left(\max\;\left[\frac{|U_{i}|+\bar{|\tilde{U_{i}}|}}{\Delta x_{i}},\frac{|V_{i}|+\bar{|\tilde{V_{i}}|}}{\Delta y_{i}},\frac{|W_{i}|}{\Delta z_{i}}\right];i=1\ldots n\right)}^{-1}$$ (2)

where *C*_max_ is the maximum allowable Courant number (set to 1.0 for InMAP), the *U*, *V*, and *W* variables are annual average wind speeds in each grid cell *i* of *n* total grid cells as defined below, $\bar{|\tilde{U_{i}}|}$ and $\bar{|\tilde{U_{i}}|}$ are annual average absolute wind speed deviations as described below, and Δ*x*, Δ*y*, and Δ*z* are the dimensions of each grid cell. With the model settings described here Δ*t* ∼ 1 min and is limited by the Courant number in the 1 km grid cells near ground level (typical annual average ground-level wind speed: 1 to 8 m s^−1^). At the top of the model domain where wind speeds are relatively fast (up to 30 m s^−1^ annual average), InMAP uses relatively large (48 km) grid cells to allow larger time steps. In contrast, in CTMs with constant-resolution grids, Δ*t* is often limited by conditions in the top grid cells rather than at ground-level, so a 1 min time step typically corresponds to a horizontal resolution grid of 10 km. The net result is a similar Δ*t* in InMAP as in a typical CTM (∼ 1 min), but with smaller ground-level grid cells in InMAP relative to in a typical CTM.

During each time step in each grid cell, InMAP first adds the flux of new emissions, accounting for plume rise from elevated sources [37] (as cited in [3]). The model then calculates how changes in pollutant concentrations are affected by physical and chemical processes including advection, turbulent mixing, atmospheric aerosol chemistry, dry deposition, and wet deposition. Each process, with the exception of the instantaneous gas- vs. particle-phase partitioning of organic, nitrate, and ammonia compounds, uses an algorithm that calculates changes in concentrations based on the concentration at the beginning of the time step rather than the concentration output by other process algorithms during the same time step. Therefore, the concentrations resulting from these steps do not depend on the order of process integration. The instantaneous gas-particle partitioning, the result of which is theoretically influenced by the order of integration, is performed last.

#### Input data

To reduce model complexity and runtime in the InMAP model itself, an InMAP preprocessor uses the output of a more comprehensive model to extract emergent atmospheric properties. Here, we use a previously published WRF-Chem model simulation [8], but the preprocessor could also be adapted for use with other models and configurations. The preprocessor makes it so that InMAP users do not need to access the CTM results directly, only the results of the preprocessor are required to run the model. InMAP uses WRF-Chem data in timesteps as output by WRF-Chem; for results here the input data timestep is once per WRF-Chem simulation hour.

Many of the chemical and physical processes important to the fate and transport of air pollution vary with the time of day and the season. A steady-state, annual-average model risks being unable to represent the results of these temporally-explicit phenomena. InMAP mitigates this potential limitation by using temporally explicit information wherever possible when calculating annual average input properties. For instance, the gas-phase oxidation of SO_2_ to SO_4_^2−^ is represented as the product of the SO_2_ concentration and a reaction rate constant, but the reaction rate constant has a non-linear dependence on temperature and on the concentration of hydroxyl radical (HO*), both of which are temporally variable. To represent the formation of particulate SO_4_ (*p*SO_4_), InMAP needs an annual average rate constant. To capture some of the effects of temporal variability, instead of calculating the rate constant using annual average values for temperature and HO*, we instead use temporally explicit temperatures, solar radiation intensities, and HO* concentrations to then calculate rate constants for every hour during the year, and then take the average of these 8760 rate-constant values. Thus, the reaction rate InMAP uses for a given grid cell is an annual-average rate, not a rate calculated using annual-average values for input parameters.

In addition to SO_2_ oxidation rates, information collected or inferred from the comprehensive model includes spatially explicit annual averages of wind vectors, eddy diffusivity and convective transport coefficients (annual average coefficients calculated using temporally explicit wind speed, temperature, pressure, friction velocity, boundary layer height, and heat flux information), dry and wet deposition rates of various pollutants (annual average rates calculated using temporally explicit wind speed, land cover, stability, and precipitation information), gas/particle phase partitioning for pollutants (described below), and parameters relevant to the calculation of emissions plume rise (annual averages of scalar windspeed; windspeed to the powers of −1, −1/3, and −1.4; temperature; and two parameters related to atmospheric stability). A full list of WRF-Chem variables used by the InMAP preprocessor is available in S1 Table.

#### Advection

The wind velocity that is responsible for advection ($\nabla \cdot (\vec{v}C)$ in Eq 1) varies at time scales smaller than can be resolved by InMAP or by comprehensive CTMs. Therefore, variables $\vec{v}$ and *C* in the advective transport term of Eq (1) are commonly split up into resolved and unresolved components using Reynolds decomposition. CTMs typically split each variable *x* into two parts: one representing the average quantity of the variable during a model timestep ($\bar{x}$) and one representing the variability of the variable during the same timestep (*x*′). For InMAP to make predictions based on annual average information, it splits each variable into three parts instead of two: $\vec{v}=\bar{\vec{v}}+\tilde{\vec{v}}+{\vec{v}}^{\prime }$ and $C=\bar{C}+\tilde{C}+C^{\prime }$, where $\bar{\vec{v}}$ and $\bar{C}$ represent annual average quantities, $\tilde{\vec{v}}$ and $\tilde{C}$ represent deviations from the annual average that are temporally resolved by the underlying CTM (WRF-Chem in this case), and ${\vec{v}}^{\prime }$ and *C*′ represent deviations that are not temporally resolved by the underlying CTM. Substituting these decomposed variables into the advection term of Eq (1) and applying the rules of Reynolds averaging yields Eq (3).

$$\nabla \cdot \left(\vec{v}C_{i}\right)=\nabla \cdot \left(\bar{\vec{v}}\bar{C_{i}}\right)+\nabla \cdot \left(\tilde{\vec{v}}\tilde{C_{i}}\right)+\nabla \cdot \left({\vec{v}}^{\prime }\tilde{C_{i}}\right)+\nabla \cdot \left(\tilde{\vec{v}}C_{i}^{\prime }\right)+\nabla \cdot \left({\vec{v}}^{\prime }C_{i}^{\prime }\right)$$ (3)

InMAP discretizes $\nabla \cdot (\bar{\vec{v}}\bar{C_{i}})$ using the upwind advection scheme shown in Eq (4):

$$\frac{\Delta C_{i}}{\Delta t}=\left\{\begin{array}{cc} \frac{\sum_{w_{j}=1}^{n_{w,i}}U_{i}C_{w_{j}}f_{w_{j}}}{\Delta x}, & \text{if}\;U_{i}\geq 0\\ \frac{\sum_{w_{j}=1}^{n_{w,i}}U_{i}C_{i}f_{w_{j}}}{\Delta x}, & \text{if}\;U_{i}<0 \end{array}\right.$$ (4)

where Δ*C*_*i*_ is the change in volume-specific pollutant concentration in grid cell *i* caused by advection between cell *i* and each cell *w*_*j*_ of *n*_*w*,*i*_ adjacent cells to the West during time step Δ*t*. Because grid resolution varies, each cell may have more than one adjacent cell in each direction. *U*_*i*_ is the annual average wind velocity vector component in the East–West direction at the interface between cells *i* and *w*_*i*_, *C*_*i*_ and *C*_*w*_*i*__ are concentrations in their respective grid cells at the beginning of the time step, *f*_*w*_*j*__ is the fraction of the edge of grid cell *i* that is touching neighbor *w*_*j*_, and Δ*x*_*i*_ is the length of the grid cell in the East–West direction. Eq (4) is repeated for neighbors to the East, to the South, to the North, above, and below cell *i*.

We chose the upwind advection scheme for its computational efficiency. A limitation of this scheme is that it is numerically diffusive, but this limitation is mitigated in InMAP because the variable resolution model grid uses smaller grid cells in high-population areas and thus limits numerical diffusion in the areas where accurate predictions are most important.

InMAP parameterizes $\nabla \cdot (\tilde{\vec{v}}\tilde{C_{i}})$ using the diffusion-like symmetrical advection scheme shown in Eq (5):

$$\frac{\Delta C_{i}}{\Delta t}=\frac{\sum_{w_{j}=1}^{n_{w,i}}\bar{|\tilde{U_{i}}|}\;\left(C_{w_{j}}-C_{i}\right)\;f_{w_{j}}}{\Delta x}$$ (5)

where $\bar{|\tilde{U_{i}}|}$ is the annual average absolute deviation of wind speed in the East–West direction, as calculated by WRF-Chem, at the interface between cells *i* and *w*_*j*_. Eq (5) is repeated for neighbors to the East, to the South, to the North, above, and below cell *i*. This scheme assumes that deviations from annual average wind velocity are symmetrical about each axis.

Finally, InMAP parameterizes $\nabla \cdot ({\vec{v}}^{\prime }C_{i}^{\prime })$ using the combined local-nonlocal mixing scheme described below. We assume that the remaining two terms in Eq (3), $\nabla \cdot ({\vec{v}}^{\prime }\tilde{C_{i}})$ and $\nabla \cdot (\tilde{\vec{v}}C_{i}^{\prime })$, account for a relatively small fraction of chemical transport. The results presented here—which show InMAP can perform reasonably without these two terms—support this assumption, but finding suitable parameterizations is an area for future research.

As shown above, to represent temporally-variable advection in an annual average modelling framework, InMAP splits advective transport into three steps, one of which is directional and two of which are symmetrical with respect to one or more axes. One result of this is that in some cases information regarding transport direction may be lost. For instance, an extreme case were wind travels from the Northwest half of the time at 2 m s^−1^ and from the Southeast the other half of the time at 2 m s^−1^ would be represented by InMAP as directional advection at 0 m s^−1^ and symmetrical advection in all horizontal directions at $\sqrt{2}\;\text{m}\;{\text{s}}^{-1}$.

For advection and mixing, InMAP assumes zero concentration-change boundary conditions at the lateral and top edges of the model domain and an impermeable boundary at the bottom edge of the domain.

#### Mixing

For mixing (i.e., pollutant transport that is not resolved by WRF-Chem; $\nabla \cdot ({\vec{v}}^{\prime }C_{i}^{\prime })$ in Eq 3) within the planetary boundary layer, we use a combined local-nonlocal closure scheme [38]. For mixing above the boundary layer and for horizontal mixing, we only consider turbulent mixing [39]. We modify a previously published relationship [38] as shown in Eq (6) to allow a variable number of adjacent cells and to include horizontal and vertical mixing.

$$m_{g,i}=\sum_{g_{j}}^{1,n_{g,i}}\;\left({\text{M2u}}_{i}C_{g_{j}}f_{g_{j}}\right)$$ (6)

$$m_{a,i}=\sum_{a_{j}}^{1,n_{a,i}}\;\left(\left[{\text{M2d}}_{a_{j}}C_{a_{j}}\frac{\Delta z_{a_{j}}}{\Delta z_{i}}-{\text{M2d}}_{i}C_{i}+\Delta z_{i}^{-1}K_{zz,a_{j}}\frac{2\left(C_{a_{j}}-C_{i}\right)}{\Delta z_{i}+\Delta z_{a_{j}}}\right]\;f_{a_{j}}\right)$$ (7)

$$m_{b,i}=\sum_{b_{j}}^{1,n_{b,i}}\;\left(\Delta z_{i}^{-1}K_{zz,b_{j}}\frac{2\;\left(C_{b_{j}}-C_{i}\right)}{\Delta z_{i}+\Delta z_{b_{j}}}f_{b_{j}}\right)$$ (8)

$$m_{w,i}=\sum_{w_{j}}^{1,n_{w,i}}\;\left(\Delta x_{i}^{-1}K_{xx,w_{j}}\frac{2\;\left(C_{w_{j}}-C_{i}\right)}{\Delta x_{i}+\Delta z_{w_{j}}}f_{w_{j}}\right)$$ (9)

$$m_{e,i}=\sum_{e_{j}}^{1,n_{e,i}}\;\left(\Delta x_{i}^{-1}K_{xx,e_{j}}\frac{2\;\left(C_{e_{j}}-C_{j}\right)}{\Delta x_{i}+\Delta x_{e_{j}}}f_{e_{j}}\right)$$ (10)

$$m_{s,i}=\sum_{s_{j}}^{1,n_{s,i}}\;\left(\Delta y_{i}^{-1}K_{yy,n_{j}}\frac{2\;\left(C_{s_{j}}-C_{i}\right)}{\Delta y_{i}+\Delta y_{s_{j}}}f_{s_{j}}\right)$$ (11)

$$m_{n,i}=\sum_{n_{j}}^{1,n_{n,i}}\;\left(\Delta y_{i}^{-1}K_{yy,n_{j}}\frac{2\;\left(C_{n_{j}}-C_{i}\right)}{\Delta y_{i}+\Delta y_{n_{j}}}f_{n_{j}}\right)$$ (12)

$$\Delta C_{i}=\left(m_{g,i}+m_{a,i}+m_{b,i}+m_{w,i}+m_{e,i}+m_{s,i}+m_{n,i}\right)\Delta t$$ (13)

In Eqs (6–13), *C*_*i*_ refers to the pollutant concentration in grid cell *i*, *g*_*j*_ refers to one of *n*_*g*,*i*_ cells at ground level directly below the cell of interest, and *b*_*j*_, *a*_*j*_, *w*_*j*_, *e*_*j*_, *s*_*j*_, and *n*_*j*_ refer to cells directly below, above, west, east, south, and north of the cell of interest, respectively. M2u and M2d are upward and downward convective mixing coefficients [38]. *K*_*zz*_ is the turbulent mixing coefficient in the vertical direction, and *K*_*xx*_ and *K*_*yy*_ are horizontal mixing coefficients. We calculate mixing coefficients (both local and nonlocal) for each time step in the WRF-Chem model output, using the boundary layer height specific to that time step, and then use the average of these values to represent mixing in InMAP.

#### Chemistry

In InMAP, total PM_2.5_ is comprised of primary PM_2.5_, which is assumed to be nonvolatile and nonreactive, and secondary PM_2.5_ which can be formed from VOCs, SO_*x*_, NO_*x*_, or NH_3_. To model the secondary formation of PM_2.5_ (*R* in Eq 1), InMAP estimates formation of particulate sulfate and ammonium using first-order chemical reaction kinetics. Partitioning between the gas and aerosol phases for nitrogen oxide, ammonia, and organic compounds (VOCs and SOA) is done assuming instantaneous adjustment to match equilibrium partitioning coefficients. Because InMAP is designed to predict the impacts of marginal changes in emissions and because the chemical relationships are nonlinear, we calculate reaction rates and partitioning coefficients for marginal changes in concentrations.

There are two main pathways from sulfur dioxide (SO_2_) gas to sulfate (SO_4_^2−^) particles: gas phase oxidation by hydroxyl radical (HO*) and aqueous phase oxidation by hydrogen peroxide (H_2_O_2_) [3]. There are no major pathways for reaction of SO_4_^2−^ back to SO_2_. After calculating an annual average overall reaction rate *k*_*S*_ for SO_2_ to SO_4_^2−^ using WRF-Chem output data and formulas for the gas phase and aqueous pathways from [3], we calculate the formation of SO_4_^2−^ particles from SO_2_ gas as in Eq (14):

$$\Delta C_{S,\text{g2p},i}=k_{S,i}C_{S,\text{g},i}\Delta t$$ (14)

where Δ*C*_S,g2p,*i*_ is the transformation of sulfur from gas to particle phase during time step Δ*t* in cell *i* and *C*_S,g,*i*_ is the gas phase concentration of sulfur at the beginning of the time step.

For NO_*x*_, NH_3_, and VOCs, the chemical reaction mechanisms governing partitioning between the gas and particle phase are more complex than the reactions driving sulfate formation. They are also reversible: gas-phase compounds can convert to aerosols and then back to gas-phase, and the direction of the reactions can vary according to the time of day and according to the season. It is not possible to directly represent these reactions in a steady-state, annual average model such as InMAP. For NO_*x*_, NH_3_, and VOCs we instead calculate an annual average partitioning coefficient *f*_p,*i*_ in grid cell *i* for marginal changes in concentrations from the WRF-Chem output data as in Eq (15):

$$f_{p,i}=\sum_{j=1}^{n}\;\left(\frac{\Delta m_{p,i,j}}{\Delta m_{p,i,j}+\Delta m_{\text{g},i,j}}\right)/n$$ (15)

where Δ*m*_p,*i*,*j*_ is change in mass in cell *i* the particle phase and Δ*m*_g,*i*_ is change in mass in the gas phase from one WRF-Chem output time step *j* to the next, and *n* is the total number of output time steps (8760). Then, we use this coefficient to calculate gas/particle partitioning in InMAP using Eqs (16) and (17):

$$C_{p,i,f}=\left(C_{\text{g},i,s}+C_{p,i,s}\right)\;f_{p,i}$$ (16)

$$C_{\text{g},i,f}=\left(C_{\text{g},i,s}+C_{p,i,s}\right)\;\left(1-f_{p,i}\right)$$ (17)

where *C*_g,*i*,*s*_, *C*_p,*i*,*s*_, *C*_g,*i*,*f*_ and *C*_p,*i*,*f*_ are gas and particle phase concentrations in cell *i* at the start *s* and end *f* of the time step. The concentration at the end of one time step is used as the concentration at the beginning of the next time step. For partitioning between VOCs and secondary organic aerosol (SOA) we only consider those VOCs that are SOA precursors as defined by [40].

#### Wet and dry deposition

We assume that dry deposition *v*_dd,*i*_ for gases in cell *i* can be represented as a function of resistances in series as in Eq (18), where *r*_*a*,*i*_ is aerodynamic resistance, *r*_*b*,*i*_ is quasi-laminar boundary layer resistance, and *r*_*c*,*i*_ is surface resistance [3]. For particles, this equation is slightly altered to account for settling velocity. We calculate an annual average dry deposition velocity for each ground-level grid cell using the output from WRF-Chem and previously published algorithms for *r*_*c*,*i*_ for gases [41, 42]. To calculate *r*_*c*,*i*_ for particles, and to calculate *r*_*a*,*i*_ and *r*_*b*,*i*_, we also use previously published algorithms [3]. We then calculate dry deposition within InMAP using Eqs (18) and (19):

$$|v_{\text{dd},i}|={\left(r_{a,i}+r_{b,i}+r_{c,i}\right)}^{-1}$$ (18)

$$\Delta C_{i}=-C_{i}v_{\text{dd},i}\frac{\Delta t}{\Delta z_{i}}$$ (19)

where *C*_*i*_ is pollutant concentration in a grid cell in the lowest model layer.

We calculate an annual average wet deposition rate *r*_wd,*i*_ for each grid cell *i* using output from WRF-Chem and a simple algorithm from the EMEP model [43] that estimates a rate of wet deposition from in-cloud and below-cloud scavenging rate as a function of cloud fraction, precipitation rate, and air density. The algorithm provides separate rate estimates for particles, SO_2_, and other gases. We then calculate wet deposition within InMAP using Eq (20):

$$\Delta C_{i}=-C_{i}r_{\text{wd},i}\Delta t$$ (20)

Dry deposition is assumed to only occur in ground-level grid cells. Wet deposition is calculated for every grid cell, with location-specific deposition rates.

### User inputs

One goal for InMAP is ease of use. The only user-specified input required by running InMAP in its native layout is a shapefile or set of shapefiles [44] containing locations of changes in annual total emissions of VOCs, SO_*x*_, NO_*x*_, NH_3_, and primary fine particulate matter (PM_2.5_). Locations can be specified as polygon, line, or point entities, and can include stack attributes which InMAP uses to calculate plume rise for elevated sources. InMAP allocates emissions from shapefiles to the corresponding model cells using area-weighting.

### Performance evaluation

InMAP provides a computationally inexpensive alternative to a CTM for calculating impacts of marginal emission changes. Therefore, its performance should be evaluated in terms of predicting marginal changes in concentrations rather than total ambient concentrations. Although the strongest evaluation would be to compare InMAP predictions to measured pollutant concentrations, long-term, nationwide measurements of the effects of marginal emissions changes on pollutant concentrations do not exist. Instead, we compare InMAP predictions for scenarios with changes in emissions to those from a CTM. It is common to evaluate air pollution sensitivity models against more complex models [21, 25]. Specifically, for our model-model evaluation we employ WRF-Chem to model 11 scenarios of emission changes that would result from the hypothetical adoption of alternative light-duty transportation technologies. These scenarios include emissions from transportation, electric generation, agriculture, and various industrial sources in proportions that vary among scenarios—since these activities have different spatial distributions, the emissions scenarios are spatially heterogeneous—resulting in total PM_2.5_ concentration changes on the order of 1%. A brief description of each emissions scenario is provided in S1–S12 Figs. Additional information regarding the emission scenarios and the associated WRF-Chem modeling can be found elsewhere [34, 45]. Below, we also compare InMAP results against an existing reduced-form model: the COBRA source-receptor matrix [26].

To explore how reliably InMAP can be expected to predict larger changes in concentrations, we separately evaluate InMAP performance in predicting measured year 2005 annual average PM_2.5_ concentrations [46]. As mentioned above, InMAP is designed to predict marginal changes in concentrations rather than total concentrations; comparing InMAP against observed values represents a use of the model that is beyond what that model was designed for. Nevertheless, we conduct and evaluate InMAP in that manner here (i.e., running it as though it were a conventional CTM rather than a model for marginal changes in emissions) to provide information on how widely applicable the model is, including its use in simulations of large changes in emissions. To compare InMAP predictions to observations, we use a previously described emissions inventory [8], with the exception that anthropogenic emissions are processed using the AEP model [47] to allow allocation of area source emissions to the InMAP spatial grid without the loss of spatial information.

Finally, to investigate InMAP’s ability to predict temporally variable pollutant transport in a steady-state framework, and its ability to predict higher-resolution pollutant spatial patterns based on lower-resolution meteorology fields, we perform an additional independent comparison of InMAP vs. WRF-Chem. To perform this comparison, we first use WRF-Chem to simulate annual average transport and fate of 100 short tons per year of emissions from a single ground-level point source of non-reactive PM_2.5_ in downtown Los Angeles, California. We run the WRF-Chem simulation over a 9801×9801 km spatial domain with 9 km, 3 km, and 1 km nested grids. Each nested grid is comprised of 33×33 horizontal grid cells and 30 vertical layers centered over downtown Los Angeles. We split up year 2005 into 8 approximately 45 day periods and simulate approximately the first 15 days of each period with WRF-Chem to approximate annual average conditions. All other aspects of the WRF-Chem configuration have been previously described [8]. We use the results from the 9 km-resolution outer WRF-Chem domain to create two versions of InMAP: one with 9 km grid cells aligning with the native WRF-Chem grid cells (“InMAP LA-9km”), and one with 1–27 km variable resolution grid cells (“InMAP LA-variable”). The 3 km- and 1 km-resolution inner WRF-Chem domains are not used during the setup of InMAP. We then compare InMAP LA-9km against the 9 km resolution WRF-Chem results and we compare InMAP LA-variable against the 1 km resolution WRF-Chem results.

We use several metrics to assess model-model and model-measurement agreement, including mean bias (MB, Eq 21), mean error (ME, Eq 22), mean fractional bias (MFB, Eq 23), mean fractional error (MFE, Eq 24), and model ratio (MR, Eq 25), as well as linear regression slope (*S*), intercept (*I*), and squared Pearson correlation coefficient (*R*^2^) values. In Eqs (21)–(25), *i* corresponds to one of *n* comparisons, and *X* and *Y* are the annual average modeled or measured values we are comparing.

$$\text{MB}=\frac{1}{n}\;\sum_{i=1}^{n}\;\left(Y_{i}-X_{i}\right)$$ (21)

$$\text{ME}=\frac{1}{n}\;\sum_{i=1}^{n}\;\left|Y_{i}-X_{i}\right|$$ (22)

$$\text{MFB}=\frac{1}{n}\;\sum_{i=1}^{n}\;\frac{2\;\left(Y_{i}-X_{i}\right)}{\left(Y_{i}+X_{i}\right)}$$ (23)

$$\text{MFE}=\frac{1}{n}\;\sum_{i=1}^{n}\;\frac{2\;\left|Y_{i}-X_{i}\right|}{\left(Y_{i}+X_{i}\right)}$$ (24)

$$\text{MR}=\frac{1}{n}\;\sum_{i=1}^{n}\;\frac{Y_{i}}{X_{i}}$$ (25)

## Results

The resulting InMAP computer model is comprised of ∼ 2000 lines of code written in the Go language [48] with an additional ∼ 2900 lines of code for preprocessing WRF-Chem output data into InMAP input data. Each InMAP model run takes approximately two hours to complete on a desktop computer with an Intel Ivybridge processor; S2 Appendix lists example simulation run times for different model settings. The preprocessor takes approximately eight hours to run on a similar desktop computer, but users will not need to run the preprocessor or obtain output from a CTM unless they are interested in a spatial or temporal domain different than the continental U.S. and year 2005. As recommended for scientific reproducibility [49], the model is freely available at http://inmap.spatialmodel.com (doi:10.5281/zenodo.60671) and is licensed under the GNU General Public License (GPL). Results here are based on InMAP version 1.2.0. Preprocessed input data and the data required to reproduce the results shown here are also freely available (doi:10.5281/zenodo.166811).

### Model to model comparison: Full US

**Fig 2:**
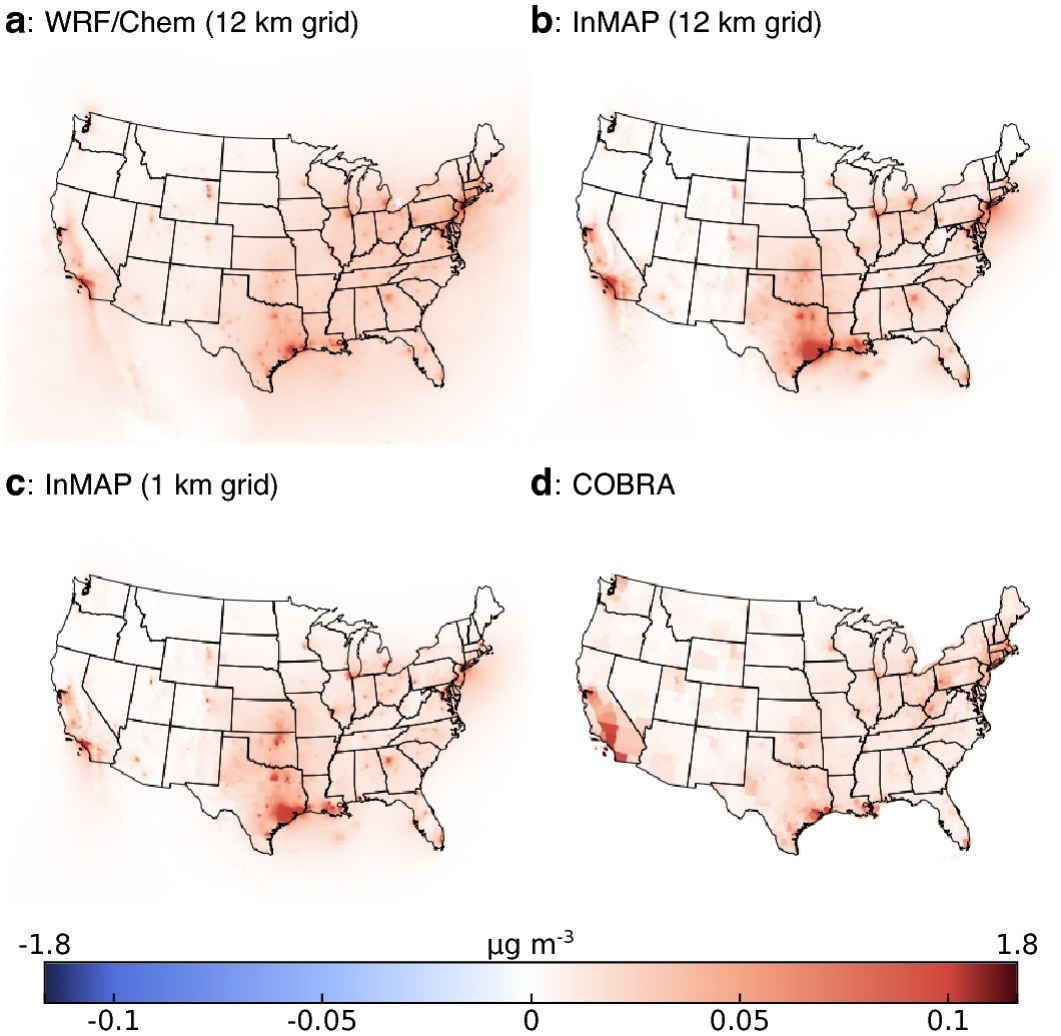
Changes in concentrations resulting from one emissions scenario as calculated by (a) WRF-Chem, (b) InMAP with a 12 km resolution grid, (c) InMAP with a 1 to 48 km variable resolution grid (i.e., a typical setup for InMAP), and (d) COBRA. For ease of viewing, there is a discontinuity at the 99th percentile of concentration values. S1–S12 Figs provide similar information for the rest of the scenarios.

Fig 2 shows WRF-Chem, InMAP, and COBRA model results for an example emissions scenario where changes in vehicle tailpipe emissions are the largest emissions source. We show two InMAP configurations: the 12 km constant-resolution grid that mirrors the grid used for WRF-Chem simulations (“InMAP 12 km”) and a variable-resolution grid for which the smallest cells are 1 km^2^ (“InMAP 1 km”). Overall, spatial patterns in concentration changes are similar in InMAP, COBRA, and WRF-Chem. In the specific example shown in Fig 2, differences in estimated concentrations are apparent in Southern California and the Gulf Coast. S1–S12 Figs show additional spatial detail comparing InMAP and WRF-Chem for this (S1 Fig) and other emissions scenarios. COBRA provides one prediction per county as can be discerned in Fig 2d where counties are large (e.g., in Southern California around Los Angeles). S1–S12 Figs contain information similar to Fig 2 for the remaining emissions scenarios investigated here, as well as corresponding performance statistics.

**Fig 3:**
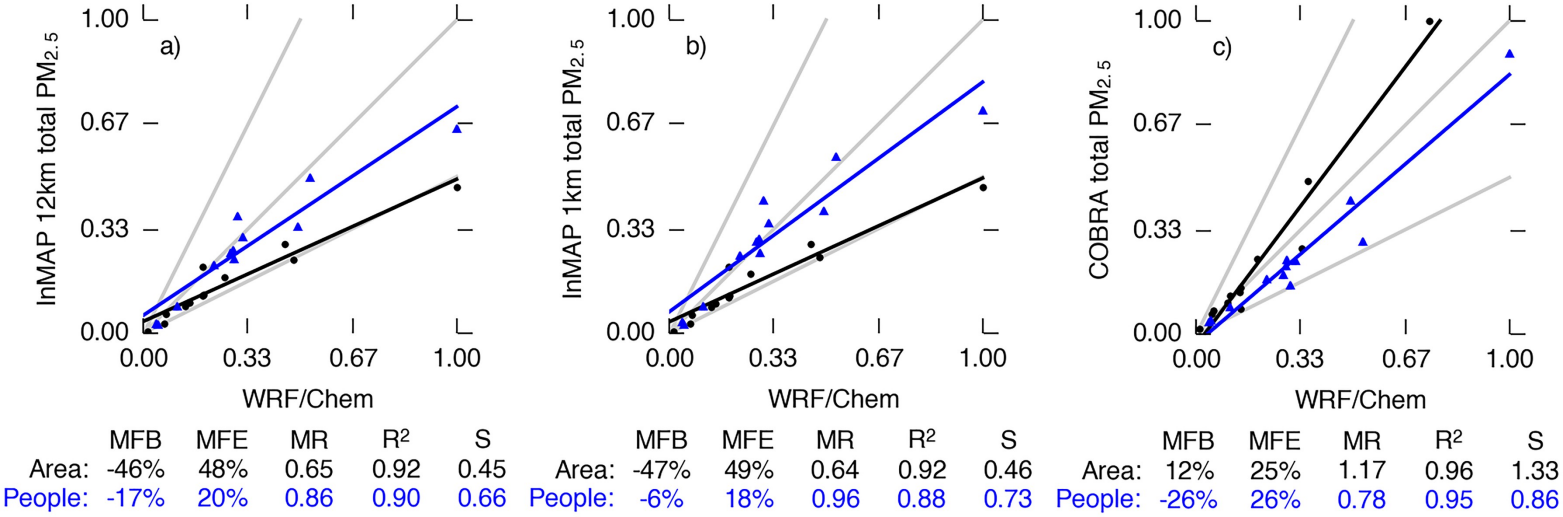
Comparison of total (primary plus secondary) area-weighted (black dots) and population-weighted (blue triangles) annual average predicted PM_2.5_ concentration change for WRF-Chem (*x* axis) and either InMAP or COBRA (*y* axis) for 11 emissions scenarios. To assist in comparison between area- and population-weighted predictions, concentrations shown here are normalized so that the largest value in each comparison equals one. The gray lines represent 1 : 1, 2 : 1, and 1 : 2 ratios between the models, and the black and blue lines represent least-squares regressions. Performance statistics for each comparison are listed below the plots. Abbreviations: MFB = mean fractional bias; MFE = mean fractional error; MR = model ratio; *R*^2^ = squared Pearson correlation coefficient; *S* = slope of regression line.

Fig 3 compares InMAP, WRF-Chem, and COBRA ground-level predictions for 12 emissions scenarios. Two sets of comparisons are shown: area-weighted (useful for understanding atmospheric processes such as mixing and removal) and population-weighted (useful for human exposures and health impacts).

InMAP 12 km reproduces the WRF-Chem predictions for changes in area-weighted concentrations with *R*^2^ = 0.92 and MFB = −46%) and in population-weighted concentrations with *R*^2^ = 0.90 and MFB = -17% (Fig 3a). InMAP 1 km performance (Fig 3b) is similar to that of InMAP 12 km. InMAP performance is not remarkably different from the existing COBRA model (Fig 3c). However, InMAP has capabilities not found in COBRA, such as predicting how pollutant concentrations vary within a county or a city and accounting for spatially variable aspects of secondary PM_2.5_ formation.

**Fig 4:**
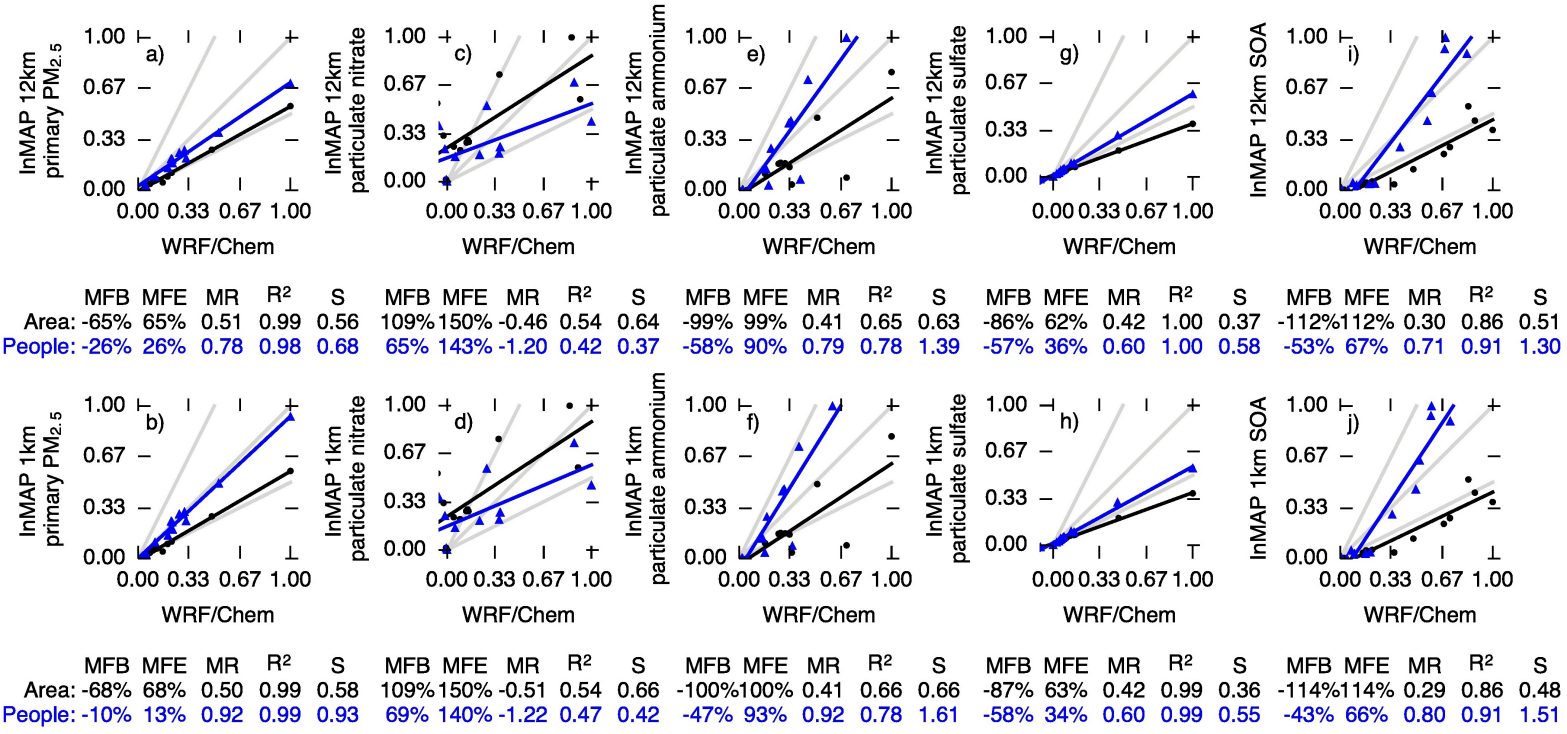
Comparison of area-weighted (black dots) and population-weighted (blue triangles) annual average predictions of changes in concentrations of PM_2.5_ subspecies between WRF-Chem (*x* axis) and InMAP (*y* axis) for 11 emissions scenarios. To assist in comparison between area- and population-weighted predictions, concentrations shown here are normalized so that the largest value in each comparison equals one. The gray lines represent InMAP: WRF-Chem ratios of 1 : 1, 2 : 1, and 1 : 2. The black and blue lines represent least-squares regressions. Performance statistics for each comparison are listed below the plots. Abbreviations: MFB = mean fractional bias; MFE = mean fractional error; MR = model ratio; *R*^2^ = squared Pearson correlation coefficient; *S* = slope of regression line.

Fig 4 compares InMAP and WRF-Chem for PM_2.5_ subgroups: primary PM_2.5_, particulate nitrate (*p*NO_3_), particulate ammonium (*p*NH_4_), particulate sulfate (*p*SO_4_), and secondary organic aerosol (SOA). InMAP primary PM_2.5_ predictions (Fig 4a and 4b) agree with WRF-Chem with *R*^2^ values of 0.98 or greater (population-weighted MFE ≤ 26%; area-weighted MFE ≤ 68%).

InMAP agreement with WRF-Chem results for *p*NO_3_ and *p*NH_4_ is the poorest of the species considered here (*R*^2^ = 0.42–0.78). *p*NO_3_ and *p*NH_4_ formation rates have large seasonal and diurnal variations, and so are more challenging to represent in a steady-state, annual average model such as InMAP.

For *p*SO_4_, InMAP predictions are well-corrolated with WRF-Chem (*R*^2^ ≥ 0.99) but tend to underpredict concentration changes (population-weighted MFB = -57%). *p*SO_4_ formation follows comparatively simple and slow-acting chemical mechanisms as described above.

For secondary organic aerosol (SOA), InMAP predictions agree relatively well with WRF-Chem for population-weighted concentration changes (MFB = −53%, *R*^2^ = 0.91). InMAP underpredicts area-weighted changes in concentrations relative to WRF-Chem (MFB ≈ −110%).

### Model to model comparison: Regional

**Fig 5:**
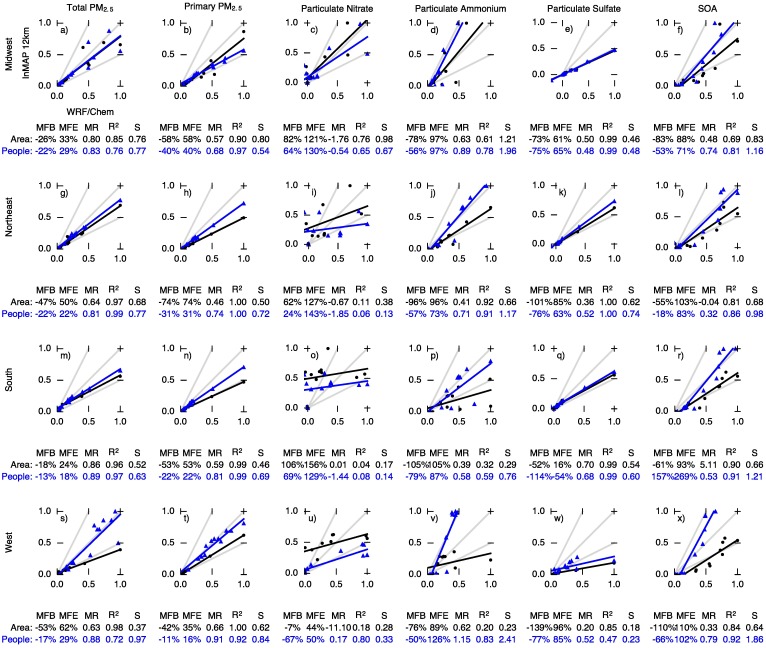
Region-specific comparisons of area-weighted (black dots) and population-weighted (blue triangles) annual average predictions of total PM_2.5_ and its subspecies between WRF-Chem (*x* axis) and InMAP (*y* axis) for 11 emissions scenarios. To assist in comparison between area- and population-weighted predictions, concentrations shown here are normalized so that the largest value in each comparison equals one. The gray lines represent InMAP: WRF-Chem ratios of 1 : 1, 2 : 1, and 1 : 2. The black and blue lines represent least-squares regressions. Performance statistics for each comparison are listed below the plots. Abbreviations: MFB = mean fractional bias; MFE = mean fractional error; MR = model ratio; *R*^2^ = squared Pearson correlation coefficient; *S* = slope of regression line.

Fig 5 shows InMAP performance by US region. (Region boundaries are in S13 Fig) Model performance is in general similar among regions. One exception is for particulate nitrate concentrations, where InMAP reproduces WRF-Chem predictions better in the Midwest (population-weighted S = 0.67) than elsewhere (population-weighted S = 0.13–0.33). This may be explained by the presence of of negative WRF-Chem predictions of *p*NO_3_ concentration changes in non-Midwest regions. These negative concentration changes are caused by interactions between PM_2.5_ subspecies that InMAP does not account for. As discussed below, these interactions are most important when changes in NO_*x*_ emissions are low, so their effect on total PM_2.5_ predictive performance is minor.

We additionally include grid-cell-specific comparisons between WRF-Chem and InMAP for the 12 emissions scenarios investigated here, as well as corresponding performance statistics, in S1–S12 Figs. Fractional performance statistics (e.g., MFB and MFE) can be highly influenced by concentrations in the lowest-concentration cells and absolute performance statistics (e.g., MB and ME) depend in part on the magnitude of emissions in each emissions scenario. We therefore focus greatest attention on population-weighted measures. Population-weighted *R*^2^ values range between 0.00 and 0.99 among scenarios and pollutant types. The lowest *R*^2^ values reflect an atypical comparison and are not a strong indication of typical model performance, for the following reason. The lowest *R*^2^ valuse are observed for *p*NO_3_ and *p*NH_4_ predictions in scenarios dominated by coal power plant emissions, where nonlinear effects related to increased SO_2_ concentrations, which are not represented in InMAP, outweigh *p*NO_3_ and *p*NH_4_ formation from NO_*x*_ and NH_3_ emissions. However, these nonlinear effects are most important when changes in *p*NO_3_ and *p*NH_4_ concentrations are low, so in these cases poor performance in predicting *p*NO_3_ and *p*NH_4_ concentrations does not necessarily adversely impact InMAP performance in predicting total PM_2.5_ concentrations.

### Model to measurement comparison

**Fig 6:**
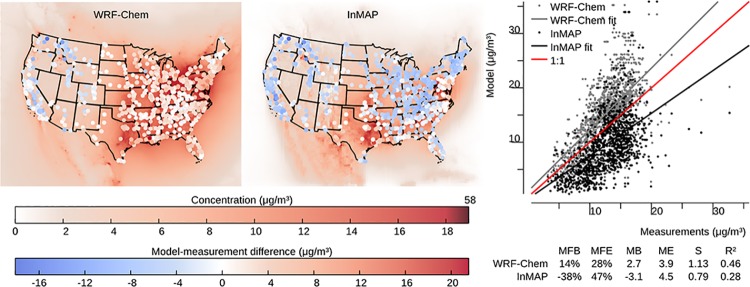
Comparison of WRF-Chem and InMAP performance in predicting annual average observed total *PM*_2.5_ concentrations. The background colors in the maps represent predicted concentrations, and the colors of the circles on the maps represent the difference between modeled and measured values at measurement locations. For the comparison shown here, on average WRF-Chem overpredicts and InMAP underpredicts as compared to observations. Abbrevations: MFB = mean fractional bias; MFE = mean fractional error; MB = mean bias; ME = mean error; MR = model ratio; *S* = slope of regression line; *R*^2^ = squared Pearson correlation coefficient.

**Fig 7:**
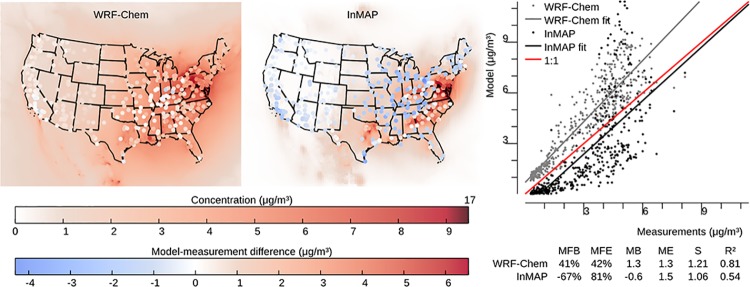
Comparison of WRF-Chem and InMAP performance in predicting annual average observed particulate SO_4_ concentrations. The background colors in the maps represent predicted concentrations, and the colors of the circles on the maps represent the difference between modeled and measured values at measurement locations. Abbrevations: MFB = mean fractional bias; MFE = mean fractional error; MB = mean bias; ME = mean error; MR = model ratio; *S* = slope of regression line; *R*^2^ = squared Pearson correlation coefficient.

**Fig 8:**
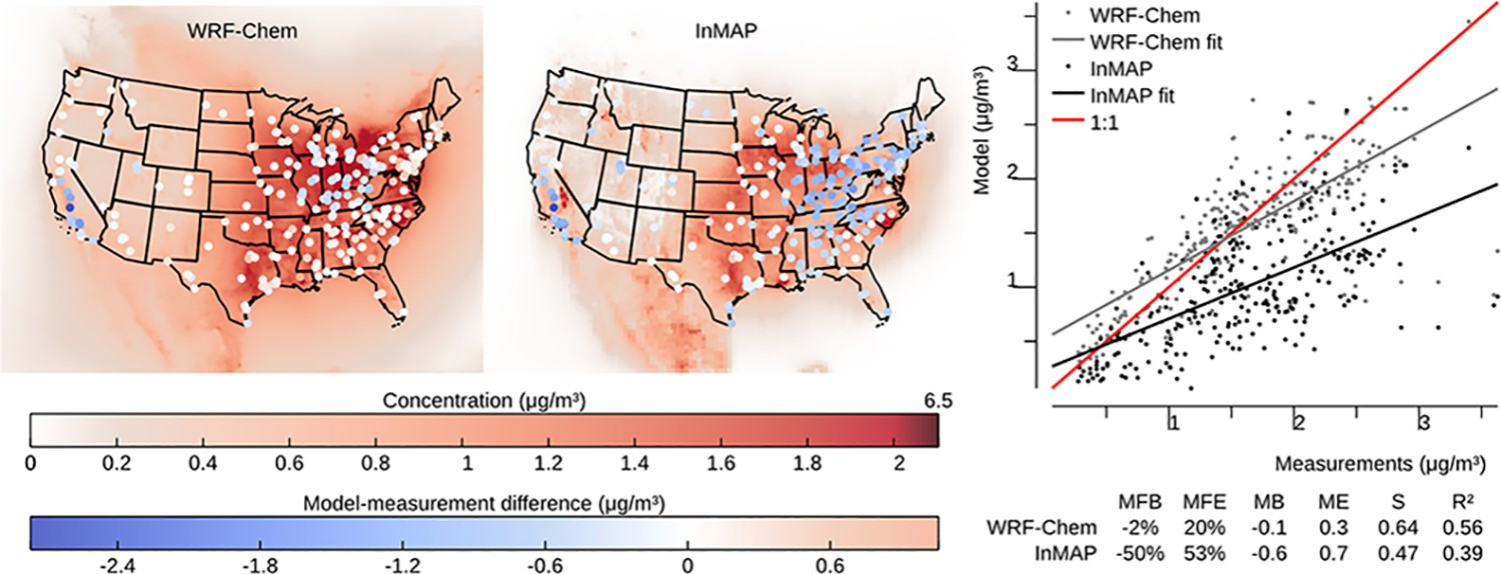
Comparison of WRF-Chem and InMAP performance in predicting annual average observed particulate NH_4_ concentrations. The background colors in the maps represent modeled concentrations, and the colors of the circles on the maps represent the difference between modeled and measured values at measurement locations. Abbrevations: MFB = mean fractional bias; MFE = mean fractional error; MB = mean bias; ME = mean error; MR = model ratio; *S* = slope of regression line; *R*^2^ = squared Pearson correlation coefficient.

**Fig 9:**
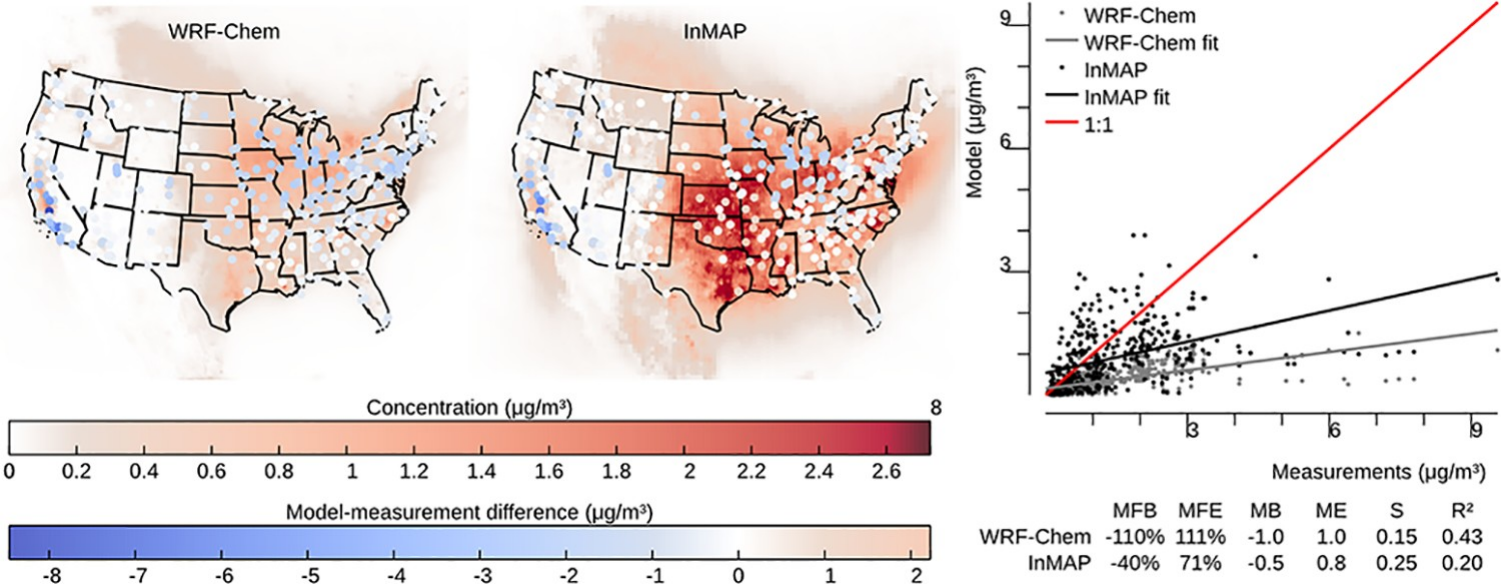
Comparison of WRF-Chem and InMAP performance in predicting annual average observed particulate NO_3_ concentrations. The background colors in the maps represent modeled concentrations, and the colors of the circles on the maps represent the difference between modeled and measured values at measurement locations. Abbrevations: MFB = mean fractional bias; MFE = mean fractional error; MB = mean bias; ME = mean error; MR = model ratio; *S* = slope of regression line; *R*^2^ = squared Pearson correlation coefficient.

InMAP is designed to model the changes in pollutant concentrations caused by marginal changes in emissions, but there are no long-term, nationwide measurements of the impacts of changes in emissions on changes in concentrations against which to evaluate InMAP directly. Therefore, we use the model-to-model comparisons described above as our main evaluation of InMAP performance. However, we also evaluate here InMAP performance in predicting overall pollutant concentrations for the year 2005. One purpose of this comparison is as a bounding estimate of how accurate InMAP would be in predicting the impacts of large changes in emissions. Figs 6–9 show the results of this comparison in terms of overall relationships between modeled and measured values and the spatial patterns in those relationships for PM_2.5_ and its subspecies. Corresponding information for gas-phase pollutants is in S14–S16 Figs. Results in Figs 6–9 for WRF-Chem refer to previously published WRF-Chem model results [8] that we use to create InMAP inputs. In general, InMAP tends to underpredict observed total PM_2.5_ concentrations (MFB = −38%; WRF-Chem MFB = 14%). However, even though InMAP is designed to predict marginal changes in concentrations rather than total concentrations, it still meets published air quality model PM_2.5_ performance criteria of MFB ≤ ±60% and MFE ≤ 75% [50] for predictions of all tested species except *p*SO_4_.

Figs 6–9 show that much of the InMAP underpredictions of total PM_2.5_ concentrations relative to observations are caused by underpredictions in *p*SO_4_. This inaccuracy in predicting observed *p*SO_4_ concentrations is not unexpected because the chemical reactions that produce *p*SO_4_ are nonlinear and InMAP is designed to predict marginal *p*SO_4_ production rather total *p*SO_4_ production. Future research could potentially re-parameterize InMAP to be a conventional (rather than marginal-change) model; that step is beyond the scope of the present article. There exist other criteria for determining model suitability [51] which could be explored in future research.

### Single source model to model comparison

**Fig 10:**
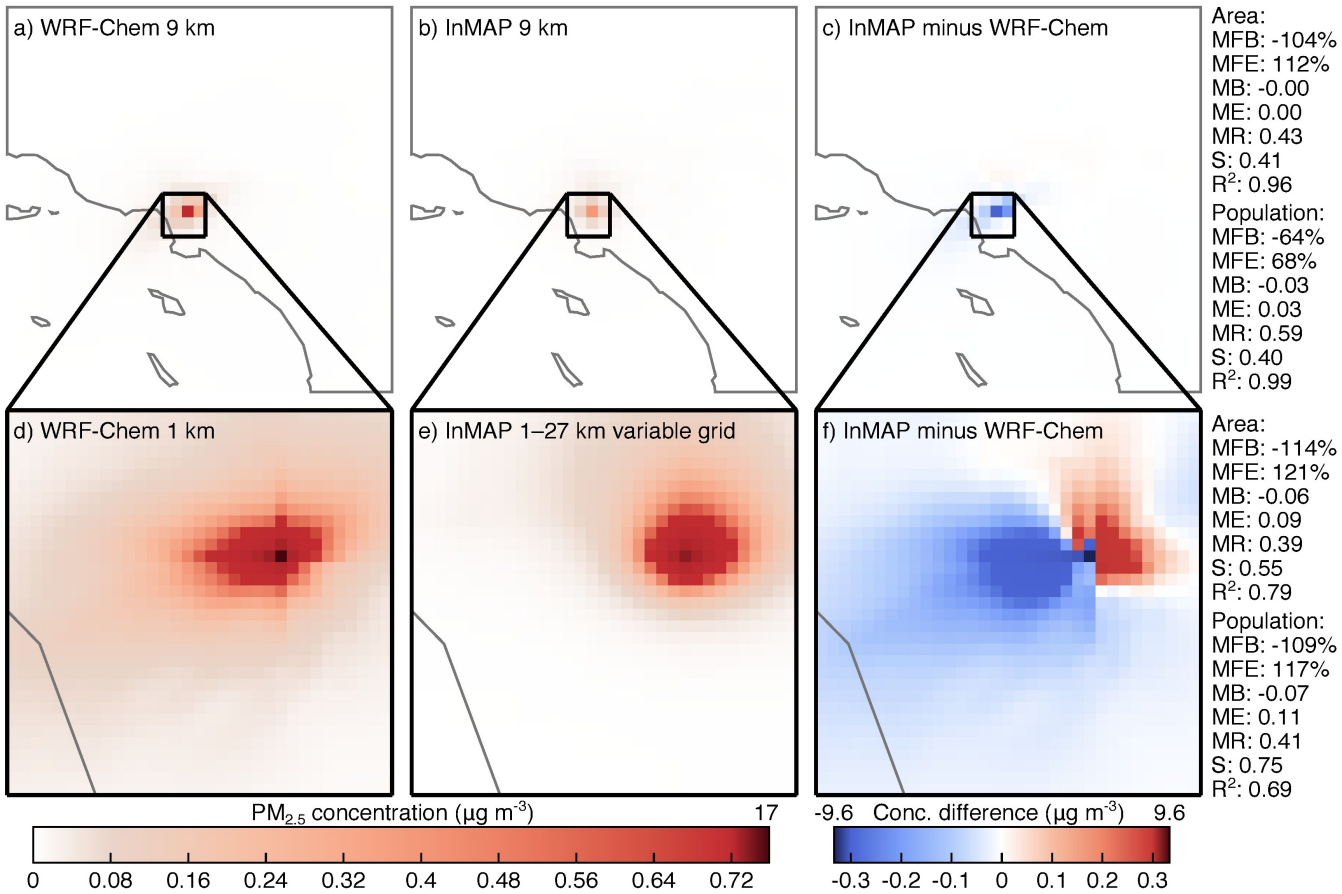
Comparison of WRF-Chem and InMAP performance in predicting the impacts of 100 tons per year of primary nonreactive PM_2.5_ emissions in Los Angeles at 9 km (panels a and b), 1 km (panel d), and 1–27 km variable (panel f) grid resolutions. InMAP predictions in panel e are based on meteorology from the 9 km-resolution WRF-Chem simulation (panel a) rather than the 1 km-resolution simulation (panel d). Panels c and f show the differences between the panels to their left. Area- and population-weighted statistics are shown on the right. Abbrevations: MFB = mean fractional bias; MFE = mean fractional error; MB = mean bias; ME = mean error; MR = model ratio; *S* = slope of regression line; *R*^2^ = squared Pearson correlation coefficient.

Fig 10 compares WRF-Chem and InMAP concentration predictions for a single ground-level point source of primary PM_2.5_ emissions. Comparisons are included for a 9 km-resolution WRF-Chem domain (Fig 10a) against a matching 9 km-resolution InMAP domain (“InMAP LA-9km”; Fig 10b), and for a nested 1 km-resolution WRF-Chem domain (Fig 10d) against a 1–27 km variable-resolution InMAP domain (“InMAP LA-variable”; Fig 10e) that was created based on the 9 km WRF-Chem results. The main difference between the two models for the 9 km domain is that WRF-Chem predicts higher concentrations in the grid cell where the emissions are located than InMAP does. One reason for this is InMAP’s use of a numerically diffusive advection scheme. The same effect can be seen in the 1 km resolution results, although these results also show that the advection solver used by WRF-Chem also creates numerical artifacts in the form of a much higher rate of transport in the exact Northward, Southward, and Westward directions from the emissions source than would generally be expected. InMAP LA-variable reproduces the spatial pattern predicted by the 1 km WRF-Chem results with MFB and MFE ≈ 100% and *R*^2^ = 0.69 − 0.79. When interpreting these results, it is important to consider that the InMAP predictions are based on lower (9 km) resolution meteorological information from WRF-Chem and should not be expected to match the WRF-Chem predictions exactly.

## Discussion

We have presented here a new air quality model for determining the human health impacts of marginal changes in pollutant emissions. InMAP is a reduced complexity model with the goal of reducing computational intensity and required user effort while minimizing losses in predictive performance. In comparisons run here, InMAP recreates WRF-Chem predictions of changes in total PM_2.5_ concentrations with population-weighted MFE and MFB < 18% and *R*^2^ ≈ 0.9. Among individual PM_2.5_ species, the best predictive performance is for primary PM_2.5_ (MFE: 26%; MFB: -26%) and the worst predictive performance is for particulate nitrate (MFE: 143%; MFB: 65%). InMAP is reduced in complexity compared to comprehensive chemical transport models but more accessible to non-specialists and more spatially detailed than other reduced-complexity national-scale air quality models. One of these existing models is the COBRA model, which we show performs similarly to the InMAP model presented here in terms of reproducing WRF-Chem changes in population-weighted average concentrations. InMAP, however, has features and capabilities that make it better suited than COBRA or other existing models for certain use cases (e.g., for simulations where it is desirable to estimate within-city, or even within-county, differences in PM_2.5_ concentrations, while also estimating long range transport of PM_2.5_ in the same simulation).

**Fig 11:**
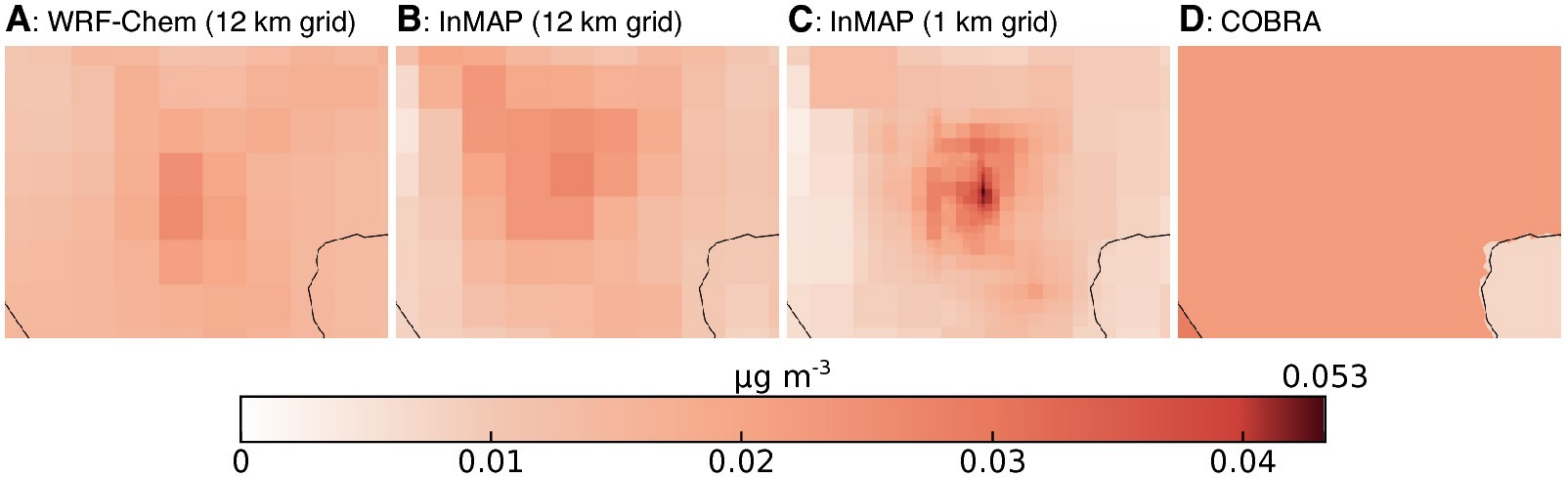
A detail view of Fig 2 centered on the city of Las Vegas. Changes in concentrations resulting from one of the emissions scenarios as calculated by **(a)** WRF-Chem, **(b)** InMAP with a 12 km resolution grid, **(c)** InMAP with a 1 to 48 km variable resolution grid, and **(d)** COBRA, which has county-level outputs. InMAP 1 km (panel c) provides the highest spatial resolution, which is important for calculating health impacts in areas with high spatial gradients in pollutant concentration and in population.

Fig 11 shows a small area of the maps in Fig 2, centered on one example urban area (Las Vegas, Nevada). COBRA represents all of the county that contains Las Vegas as having the same PM_2.5_ concentration, so most of the map is only one color. WRF-Chem, as configured here, is able to resolve differences in pollutant levels at a 12 km scale for the contiguous US (If the size of the total spatial domain were decreased to only include the area surrounding Las Vegas, WRF-Chem could resolve differences at a ∼ 1–4 km scale.) InMAP is unique among existing models in that it can model changes in pollutant concentrations across the entire contiguous US with 1 km spatial resolution in all high-population areas, all in a single model run.

The ability to resolve differences in pollution concentrations within urban areas is important for certain types of analyses, such as those that seek to determine how pollution exposure differs among demographic groups (environmental justice) or neighborhoods.

InMAP is much less computationally intensive than are CTMs. For example, InMAP 1 km produces the results for each of the scenarios shown here in ∼ 2 hr on a current desktop computer, requiring a factor of ∼ 15 000 less computational power than was required to produce the WRF-Chem results shown here. S2 Appendix lists example simulation run times for different model settings. This computational speed-up makes possible uncertainty, sensitivity, and scenario analyses that could not be attempted with WRF-Chem or other comprehensive chemical transport models.

Limitations of InMAP include the following. Model performance is better for population-weighted primary PM_2.5_, *p*SO_4_, and SOA concentrations (*R*^2^ ≥ 0.9) than for changes in *p*NO_3_ and *p*NH_4_ concentrations (*R*^2^ ∼ 0.4–0.8). The setup and testing of InMAP has mainly considered SOA formed from anthropogenic sources; further testing is needed to determine InMAP performance in predicting impacts of biogenic VOC emissions. Additional testing could be useful to further evaluate the accuracy of InMAP’s high-resolution urban area predictions against other high-resolution model simulations or measurements. At present, InMAP does not predict concentrations of ground-level ozone (O_3_), which is considered the distant-second largest source of human health burden from air pollution after PM_2.5_ [1, 2]. Additionally, InMAP performance is better for population-weighted metrics (e.g., for health studies, exposure, or environmental justice) than for area-weighted metrics (e.g., for understanding “average atmospheric” processes).

A future version of InMAP, including more comprehensive mechanisms for gas- and aerosol-phase chemistry and iterating through diurnal cycles representative of each season of the year instead of using annual average information, could potentially ameliorate many of these limitations, and would have the added benefit of allowing the prediction of concentrations of a larger number of pollutants. This approach would by necessity be more computationally intensive than the current version and require more user input information, so increased predictive power may come at the expense of ease, speed, and flexibility. A future version of InMAP could use distributed-memory parallelization or cloud computing to minimize the impact to users of any increased computational intensity. Future development is also planned to allow the preprocessor to accept output from models other than WRF-Chem, such as GEOS-Chem and CAMx.

InMAP is designed to be readily adapted to different spatial and temporal domains. This can be done by obtaining output from a CTM for the desired domain and processing it with the InMAP preprocessor. (An evaluation of model accuracy in the new domain would also be recommended.) By producing an air quality model that is computationally inexpensive to operate, relatively easily adaptable to new geographical regions, able to be operated with a moderate level of specialist knowledge, we hope to make air quality modeling more widespread, easier, and more accessible to scientists, policymakers, and concerned citizens worldwide.

## Supporting information

S1 Fig Annual average increases in pollutant concentrations caused by an emission scenario with on-road emissions from gasoline powered vehicles as the largest emissions source, as predicted by WRF-Chem (first row) and InMAP with a 12 km resolution grid (second row), as well as the difference between the two models (third row). Colors in the first two rows correspond to the legend on the left and colors in the third row correspond to the legend on the right. For ease of viewing, there is a discontinuity at the 99th percentile of concentration values in each color scale. Abbrevations: MFB = mean fractional bias; MFE = mean fractional error; MB = mean bias; ME = mean error; *S* = slope of regression line; *R*^2^ = squared Pearson correlation coefficient.
(TIF)

S2 Fig Annual average increases in pollutant concentrations caused by an emission scenario with on-road emissions from hybrid gasoline-electric powered vehicles as the largest emissions source, as predicted by WRF-Chem (first row) and InMAP with a 12 km resolution grid (second row), as well as the difference between the two models (third row). Colors in the first two rows correspond to the legend on the left and colors in the third row correspond to the legend on the right. For ease of viewing, there is a discontinuity at the 99th percentile of concentration values in each color scale. Abbrevations: MFB = mean fractional bias; MFE = mean fractional error; MB = mean bias; ME = mean error; *S* = slope of regression line; *R*^2^ = squared Pearson correlation coefficient.
(TIF)

S3 Fig Annual average increases in pollutant concentrations caused by an emission scenario with on-road emissions from diesel powered vehicles as the largest emissions source, as predicted by WRF-Chem (first row) and InMAP with a 12 km resolution grid (second row), as well as the difference between the two models (third row). Colors in the first two rows correspond to the legend on the left and colors in the third row correspond to the legend on the right. For ease of viewing, there is a discontinuity at the 99th percentile of concentration values in each color scale. Abbrevations: MFB = mean fractional bias; MFE = mean fractional error; MB = mean bias; ME = mean error; *S* = slope of regression line; *R*^2^ = squared Pearson correlation coefficient.
(TIF)

S4 Fig Annual average increases in pollutant concentrations caused by an emission scenario with on-road emissions from Compressed Natural Gas (CNG) powered vehicles as the largest emissions source, as predicted by WRF-Chem (first row) and InMAP with a 12 km resolution grid (second row), as well as the difference between the two models (third row). Colors in the first two rows correspond to the legend on the left and colors in the third row correspond to the legend on the right. For ease of viewing, there is a discontinuity at the 99th percentile of concentration values in each color scale. Abbrevations: MFB = mean fractional bias; MFE = mean fractional error; MB = mean bias; ME = mean error; *S* = slope of regression line; *R*^2^ = squared Pearson correlation coefficient.
(TIF)

S5 Fig Annual average increases in pollutant concentrations caused by an emission scenario with industrial emissions, agricultural emissions, and on-road emissions from ethanol powered vehicles as the largest emissions sources, as predicted by WRF-Chem (first row) and InMAP with a 12 km resolution grid (second row), as well as the difference between the two models (third row). Colors in the first two rows correspond to the legend on the left and colors in the third row correspond to the legend on the right. For ease of viewing, there is a discontinuity at the 99th percentile of concentration values in each color scale. Abbrevations: MFB = mean fractional bias; MFE = mean fractional error; MB = mean bias; ME = mean error; *S* = slope of regression line; *R*^2^ = squared Pearson correlation coefficient.
(TIF)

S6 Fig Annual average increases in pollutant concentrations caused by an emission scenario with industrial emissions, agricultural emissions, and on-road emissions from ethanol powered vehicles as the largest emissions sources, as predicted by WRF-Chem (first row) and InMAP with a 12 km resolution grid (second row), as well as the difference between the two models (third row). Colors in the first two rows correspond to the legend on the left and colors in the third row correspond to the legend on the right. For ease of viewing, there is a discontinuity at the 99th percentile of concentration values in each color scale. Abbrevations: MFB = mean fractional bias; MFE = mean fractional error; MB = mean bias; ME = mean error; *S* = slope of regression line; *R*^2^ = squared Pearson correlation coefficient.
(TIF)

S7 Fig Annual average increases in pollutant concentrations caused by an emission scenario with emissions from coal- and natural gas-powered electric generation and from coal mining as the largest emissions sources, as predicted by WRF-Chem (first row) and InMAP with a 12 km resolution grid (second row), as well as the difference between the two models (third row). Colors in the first two rows correspond to the legend on the left and colors in the third row correspond to the legend on the right. For ease of viewing, there is a discontinuity at the 99th percentile of concentration values in each color scale. Abbrevations: MFB = mean fractional bias; MFE = mean fractional error; MB = mean bias; ME = mean error; *S* = slope of regression line; *R*^2^ = squared Pearson correlation coefficient.
(TIF)

S8 Fig Annual average pollutant concentrations caused by an emission scenario with emissions from coal-powered electric generation and from coal mining as the largest emissions sources, as predicted by WRF-Chem (first row) and InMAP with a 12 km resolution grid (second row), as well as the difference between the two models (third row). Colors in the first two rows correspond to the legend on the left and colors in the third row correspond to the legend on the right. For ease of viewing, there is a discontinuity at the 99th percentile of concentration values in each color scale. Abbrevations: MFB = mean fractional bias; MFE = mean fractional error; MB = mean bias; ME = mean error; *S* = slope of regression line; *R*^2^ = squared Pearson correlation coefficient.
(TIF)

S9 Fig Annual average pollutant concentrations caused by an emission scenario with emissions from natural-gas powered electric generation and natural gas extraction and processing as the largest emissions sources, as predicted by WRF-Chem (first row) and InMAP with a 12 km resolution grid (second row), as well as the difference between the two models (third row). Colors in the first two rows correspond to the legend on the left and colors in the third row correspond to the legend on the right. For ease of viewing, there is a discontinuity at the 99th percentile of concentration values in each color scale. Abbrevations: MFB = mean fractional bias; MFE = mean fractional error; MB = mean bias; ME = mean error; *S* = slope of regression line; *R*^2^ = squared Pearson correlation coefficient.
(TIF)

S10 Fig Annual average pollutant concentrations caused by an emission scenario with emissions from agricultural sources and from biomass-powered electric generation as the largest emissions sources, as predicted by WRF-Chem (first row) and InMAP with a 12 km resolution grid (second row), as well as the difference between the two models (third row). Colors in the first two rows correspond to the legend on the left and colors in the third row correspond to the legend on the right. For ease of viewing, there is a discontinuity at the 99th percentile of concentration values in each color scale. Abbrevations: MFB = mean fractional bias; MFE = mean fractional error; MB = mean bias; ME = mean error; *S* = slope of regression line; *R*^2^ = squared Pearson correlation coefficient.
(TIF)

S11 Fig Annual average pollutant concentrations caused by an emission scenario with on-road emissions from vehicle brake and tire wear as the only emissions source, as predicted by WRF-Chem (first row) and InMAP with a 12 km resolution grid (second row), as well as the difference between the two models (third row). Colors in the first two rows correspond to the legend on the left and colors in the third row correspond to the legend on the right. For ease of viewing, there is a discontinuity at the 99th percentile of concentration values in each color scale. Abbrevations: MFB = mean fractional bias; MFE = mean fractional error; MB = mean bias; ME = mean error; *S* = slope of regression line; *R*^2^ = squared Pearson correlation coefficient.
(TIF)

S12 Fig Annual average pollutant concentrations caused by an emission scenario with emissions from mineral extraction and electricity production as the largest emissions sources, as predicted by WRF-Chem (first row) and InMAP with a 12 km resolution grid (second row), as well as the difference between the two models (third row). Colors in the first two rows correspond to the legend on the left and colors in the third row correspond to the legend on the right. For ease of viewing, there is a discontinuity at the 99th percentile of concentration values in each color scale. Abbrevations: MFB = mean fractional bias; MFE = mean fractional error; MB = mean bias; ME = mean error; *S* = slope of regression line; *R*^2^ = squared Pearson correlation coefficient.
(TIF)

S13 Fig Boundaries of US regions used in this article. (TIF)

S14 Fig Comparison of WRF-Chem and InMAP performance in predicting annual average observed SO_*x*_ concentrations. The background colors in the maps represent predicted concentrations, and the colors of the circles on the maps represent the difference between modeled and measured values at measurement locations. Abbrevations: MFB = mean fractional bias; MFE = mean fractional error; MB = mean bias; ME = mean error; MR = model ratio; *S* = slope of regression line; *R*^2^ = squared Pearson correlation coefficient.
(TIF)

S15 Fig Comparison of WRF-Chem and InMAP performance in predicting annual average observed NH_3_ concentrations. The background colors in the maps represent modeled concentrations, and the colors of the circles on the maps represent the difference between modeled and measured values at measurement locations. Abbrevations: MFB = mean fractional bias; MFE = mean fractional error; MB = mean bias; ME = mean error; MR = model ratio; *S* = slope of regression line; *R*^2^ = squared Pearson correlation coefficient.
(TIF)

S16 Fig Comparison of WRF-Chem and InMAP performance in predicting annual average observed NO_*x*_ concentrations. The background colors in the maps represent modeled concentrations, and the colors of the circles on the maps represent the difference between modeled and measured values at measurement locations. Abbrevations: MFB = mean fractional bias; MFE = mean fractional error; MB = mean bias; ME = mean error; MR = model ratio; *S* = slope of regression line; *R*^2^ = squared Pearson correlation coefficient.
(TIF)

S1 Appendix A review of existing reduced-complexity air quality models. (PDF)

S2 Appendix A description of the spatial discretization algorithm and the computational time required to run the model with different spatial grid settings. (PDF)

S1 Table The names of WRF-Chem variables used by the InMAP preprocessor and their descriptions. (PDF)
