# Supplementary figures and images for: InMAP: A model for air pollution interventions
Source: PLoS One. 2017 Apr 19;12(4):e0176131. doi: 10.1371/journal.pone.0176131 (PMC5397056; doi:10.1371/journal.pone.0176131)

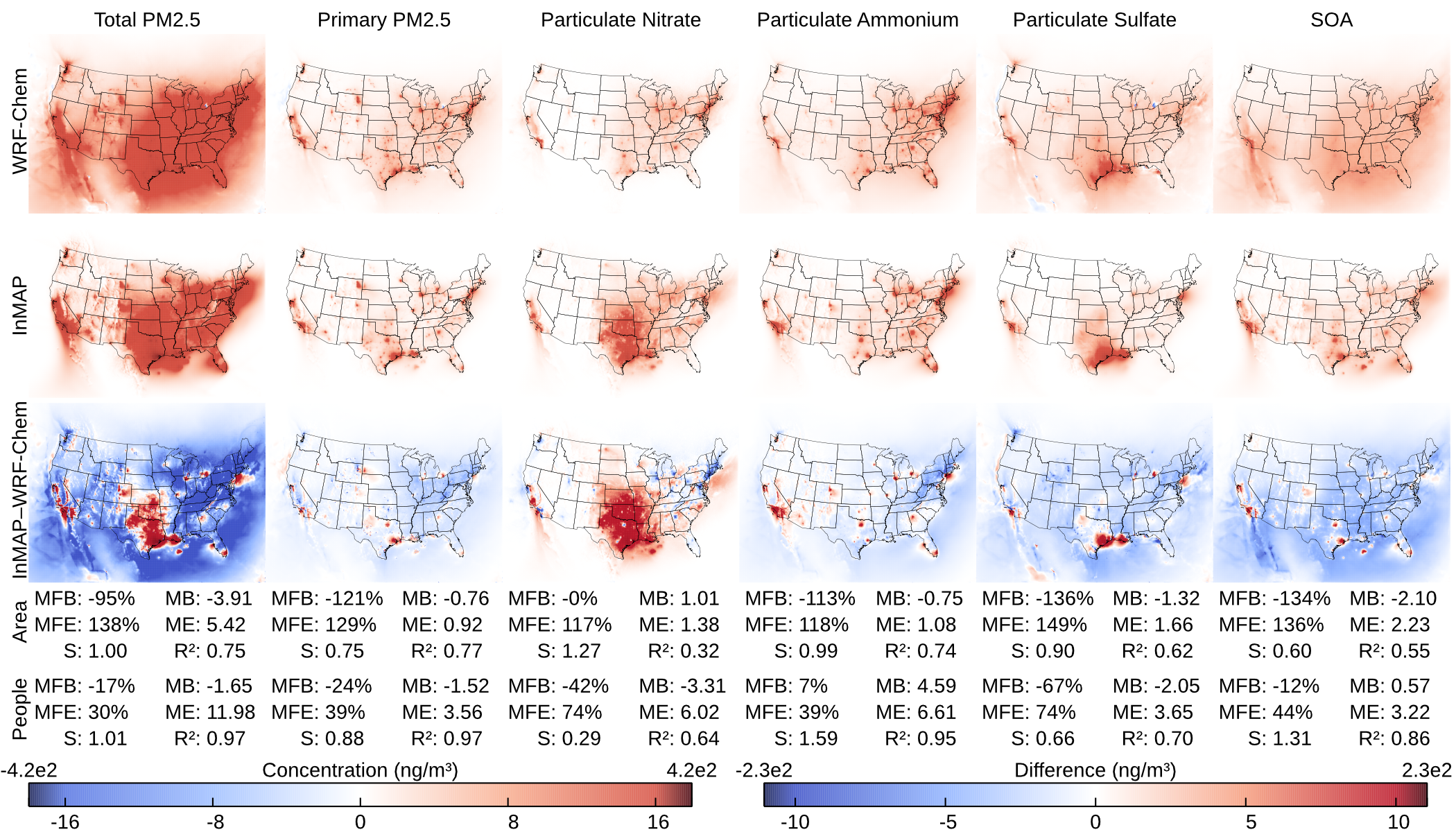

Supplement: S1 Fig — Colors in the first two rows correspond to the legend on the left and colors in the third row correspond to the legend on the right. For ease of viewing, there is a discontinuity at the 99th percentile of concentration values in each color scale. Abbrevations: MFB = mean fractional bias; MFE = mean fractional error; MB = mean bias; ME = mean error; S = slope of regression line; R2 = squared Pearson correlation coefficient. (TIF) [file pone.0176131.s001.tif]

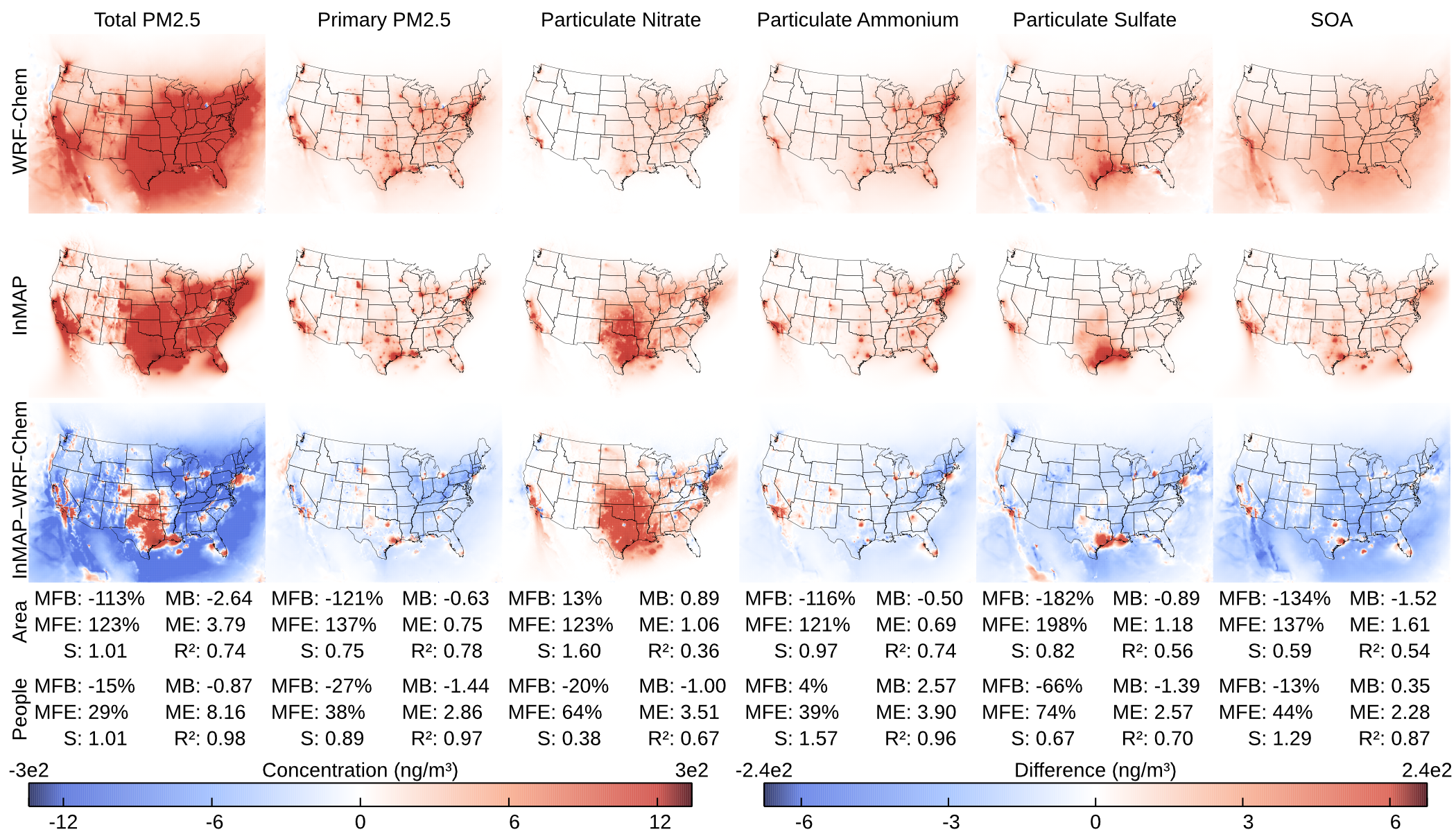

Supplement: S2 Fig — Colors in the first two rows correspond to the legend on the left and colors in the third row correspond to the legend on the right. For ease of viewing, there is a discontinuity at the 99th percentile of concentration values in each color scale. Abbrevations: MFB = mean fractional bias; MFE = mean fractional error; MB = mean bias; ME = mean error; S = slope of regression line; R2 = squared Pearson correlation coefficient. (TIF) [file pone.0176131.s002.tif]

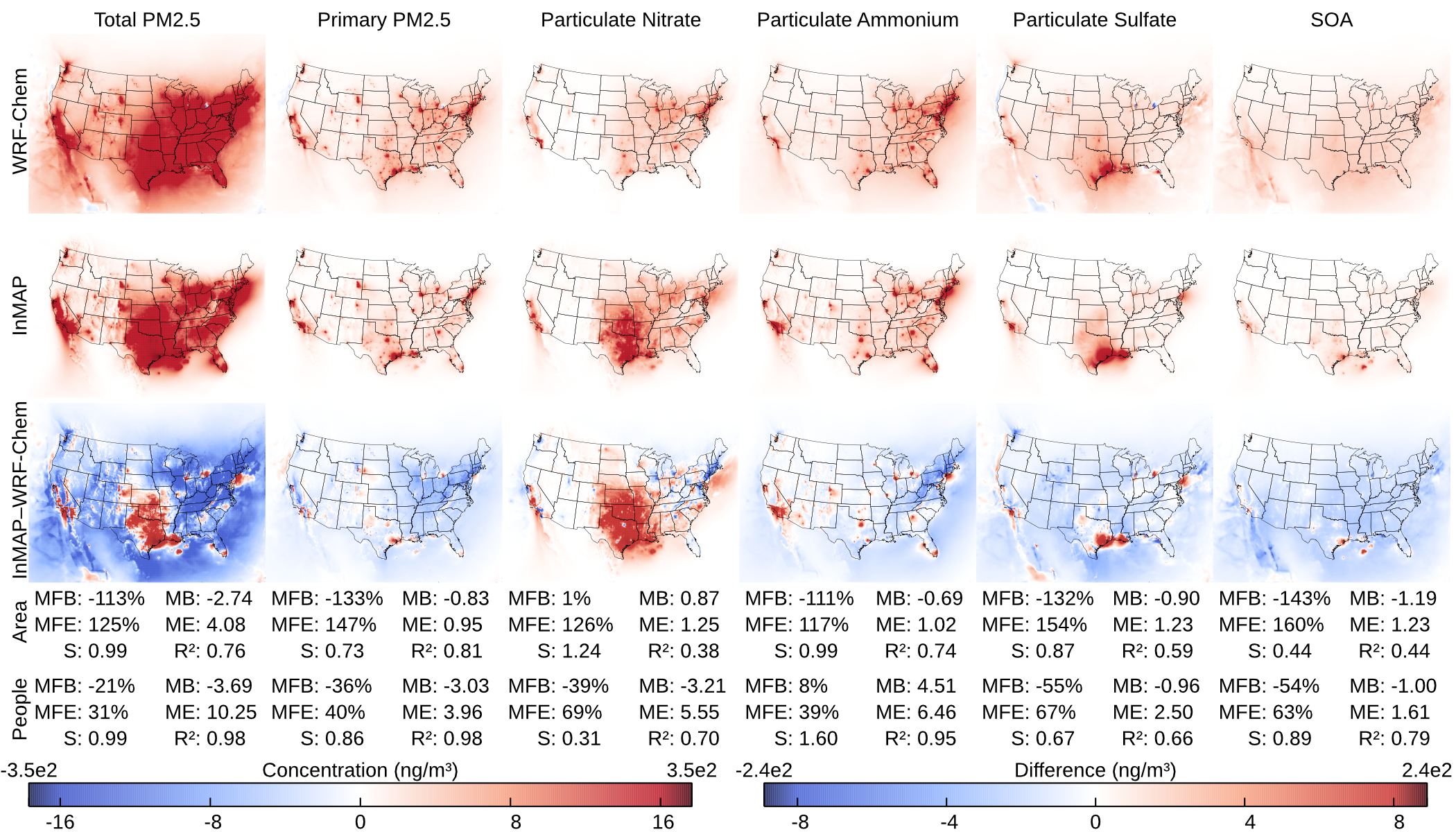

Supplement: S3 Fig — Colors in the first two rows correspond to the legend on the left and colors in the third row correspond to the legend on the right. For ease of viewing, there is a discontinuity at the 99th percentile of concentration values in each color scale. Abbrevations: MFB = mean fractional bias; MFE = mean fractional error; MB = mean bias; ME = mean error; S = slope of regression line; R2 = squared Pearson correlation coefficient. (TIF) [file pone.0176131.s003.tif]

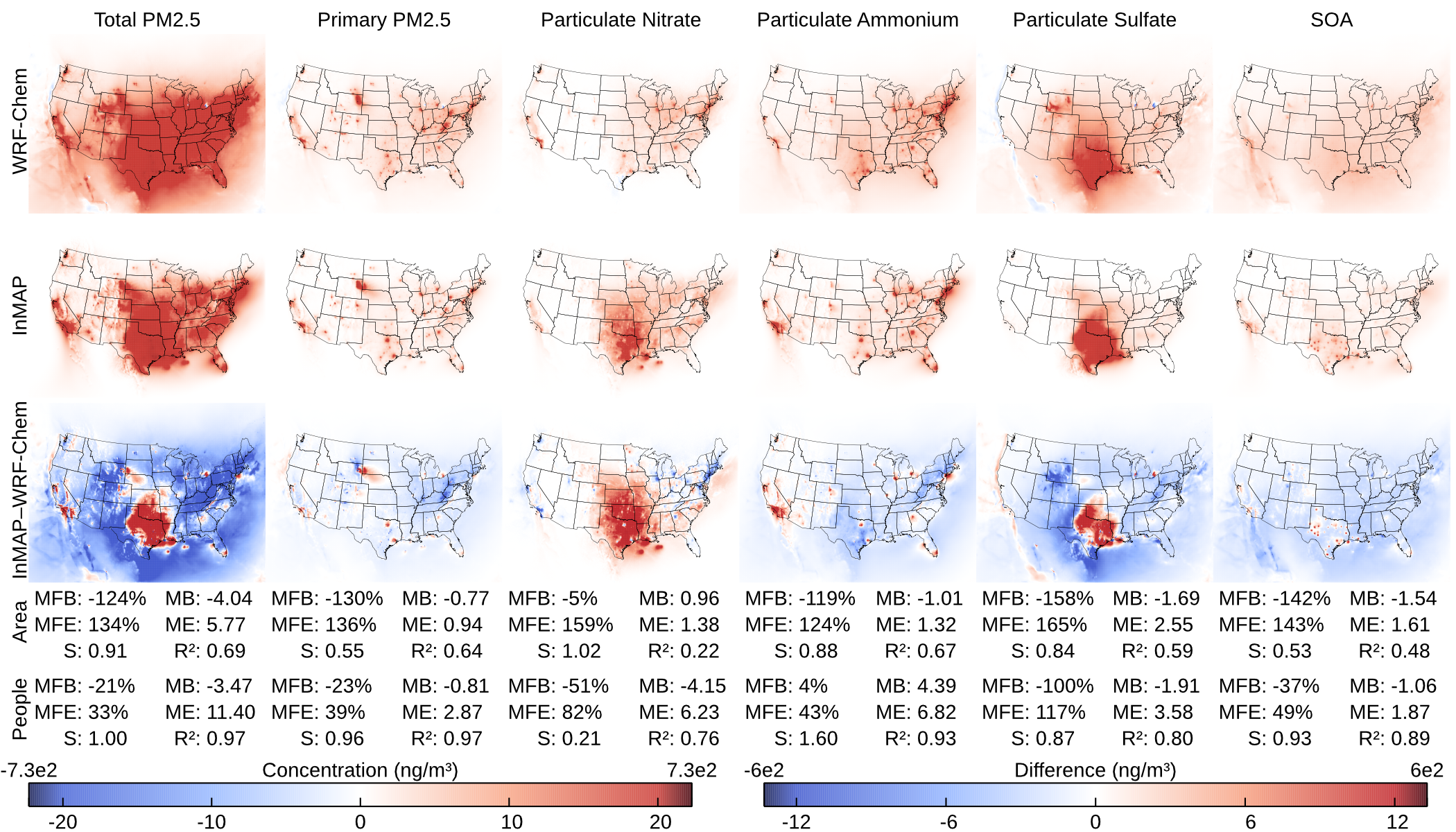

Supplement: S4 Fig — Colors in the first two rows correspond to the legend on the left and colors in the third row correspond to the legend on the right. For ease of viewing, there is a discontinuity at the 99th percentile of concentration values in each color scale. Abbrevations: MFB = mean fractional bias; MFE = mean fractional error; MB = mean bias; ME = mean error; S = slope of regression line; R2 = squared Pearson correlation coefficient. (TIF) [file pone.0176131.s004.tif]

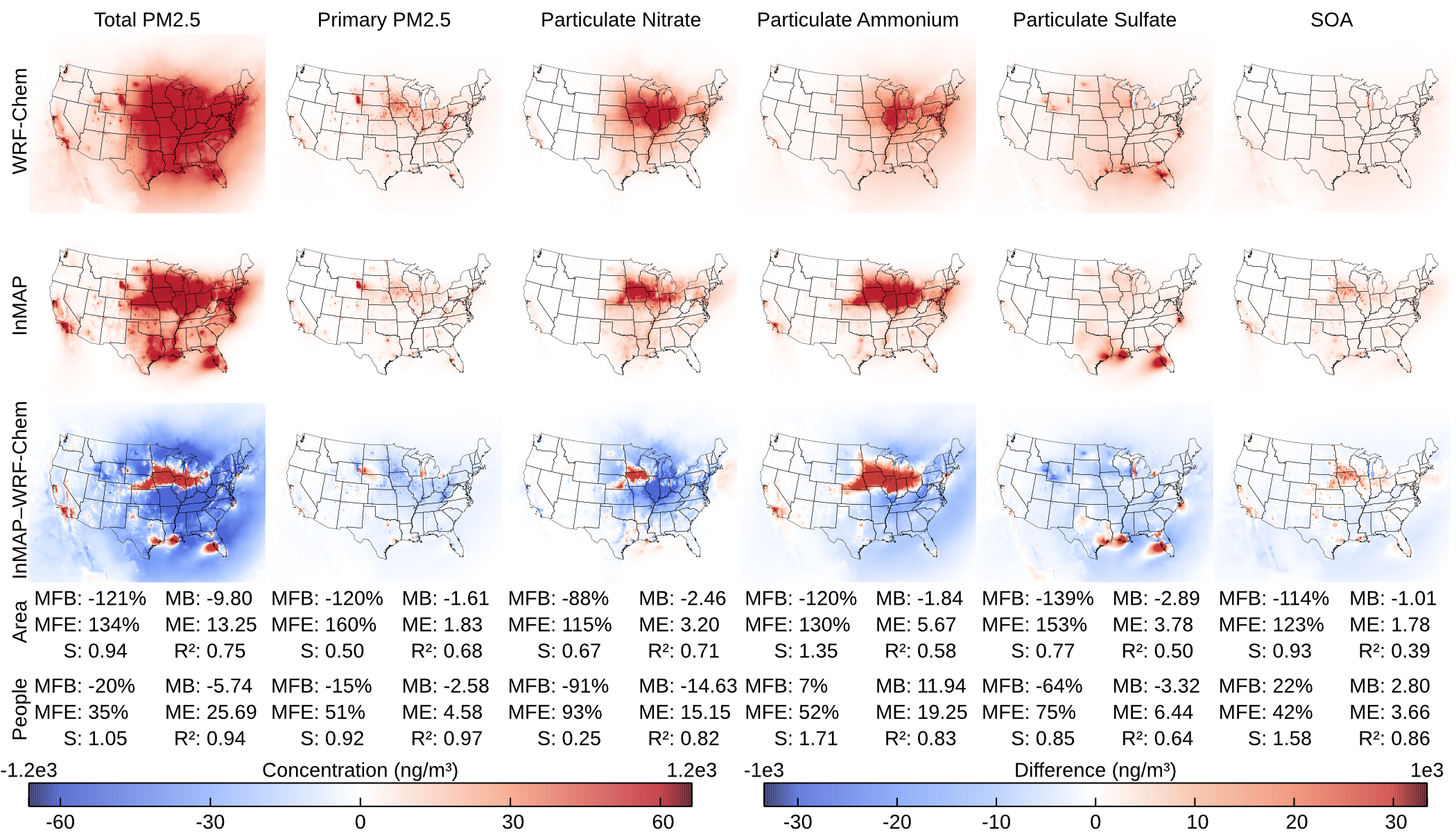

Supplement: S5 Fig — Colors in the first two rows correspond to the legend on the left and colors in the third row correspond to the legend on the right. For ease of viewing, there is a discontinuity at the 99th percentile of concentration values in each color scale. Abbrevations: MFB = mean fractional bias; MFE = mean fractional error; MB = mean bias; ME = mean error; S = slope of regression line; R2 = squared Pearson correlation coefficient. (TIF) [file pone.0176131.s005.tif]

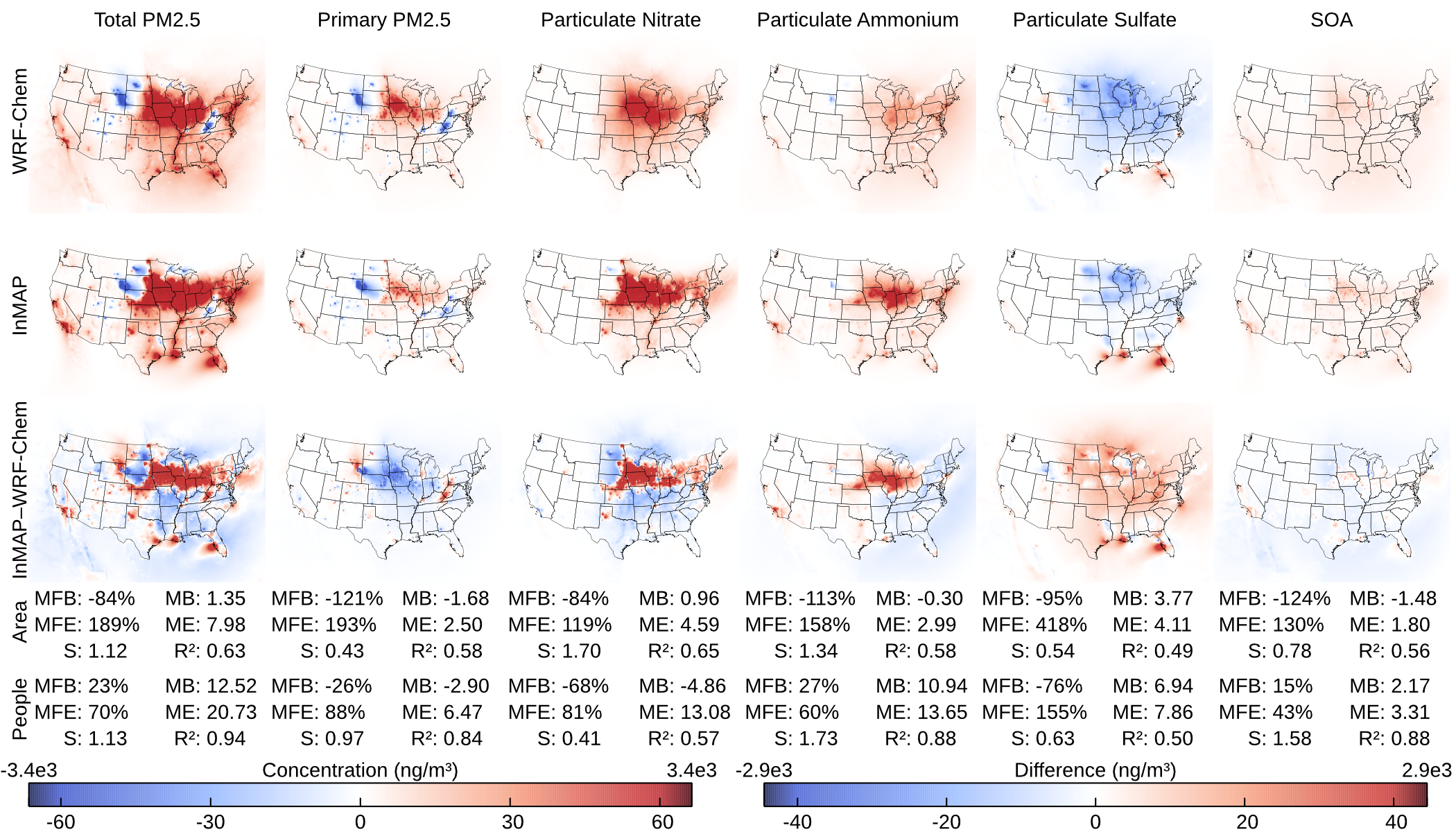

Supplement: S6 Fig — Colors in the first two rows correspond to the legend on the left and colors in the third row correspond to the legend on the right. For ease of viewing, there is a discontinuity at the 99th percentile of concentration values in each color scale. Abbrevations: MFB = mean fractional bias; MFE = mean fractional error; MB = mean bias; ME = mean error; S = slope of regression line; R2 = squared Pearson correlation coefficient. (TIF) [file pone.0176131.s006.tif]

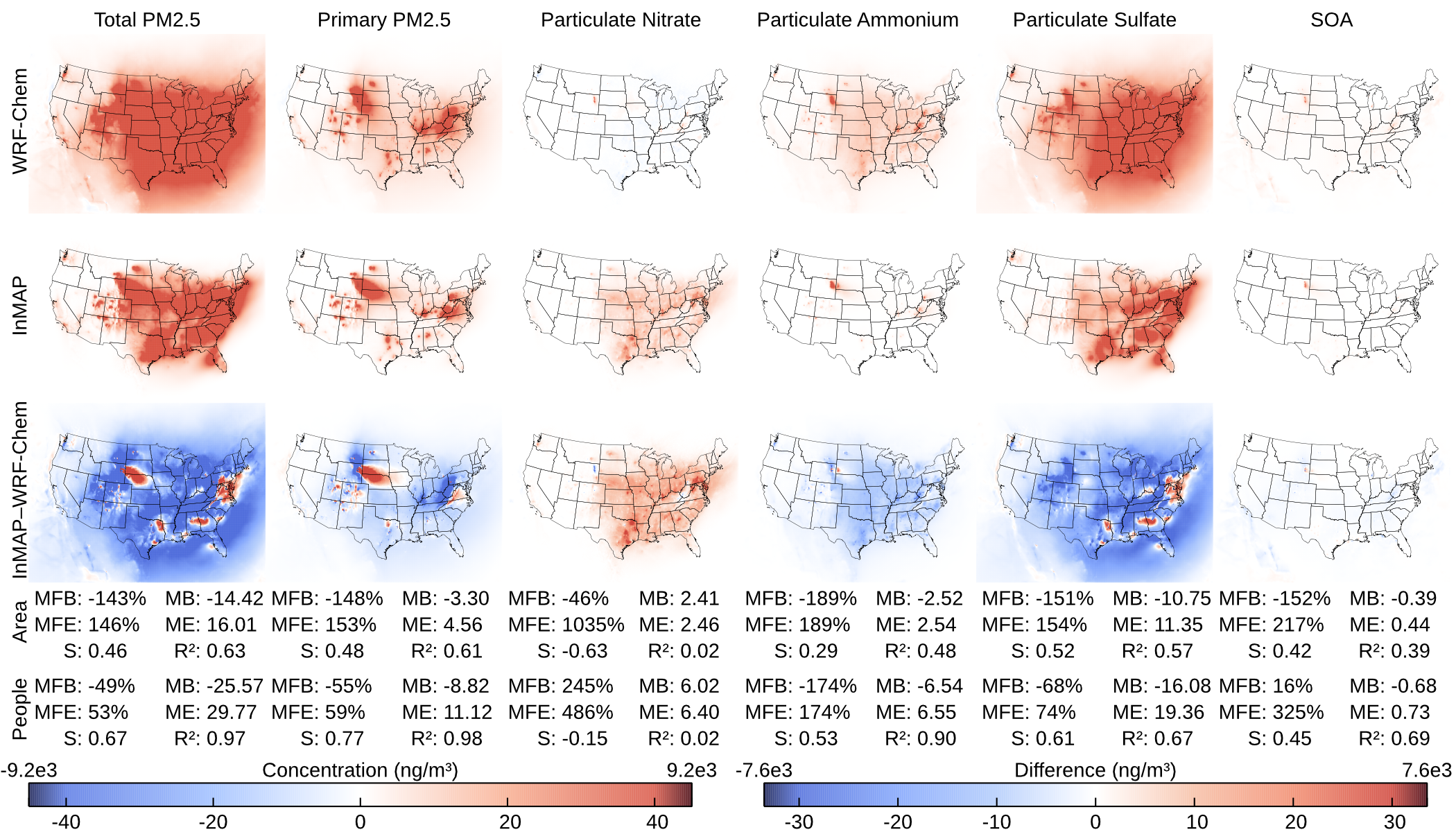

Supplement: S7 Fig — Colors in the first two rows correspond to the legend on the left and colors in the third row correspond to the legend on the right. For ease of viewing, there is a discontinuity at the 99th percentile of concentration values in each color scale. Abbrevations: MFB = mean fractional bias; MFE = mean fractional error; MB = mean bias; ME = mean error; S = slope of regression line; R2 = squared Pearson correlation coefficient. (TIF) [file pone.0176131.s007.tif]

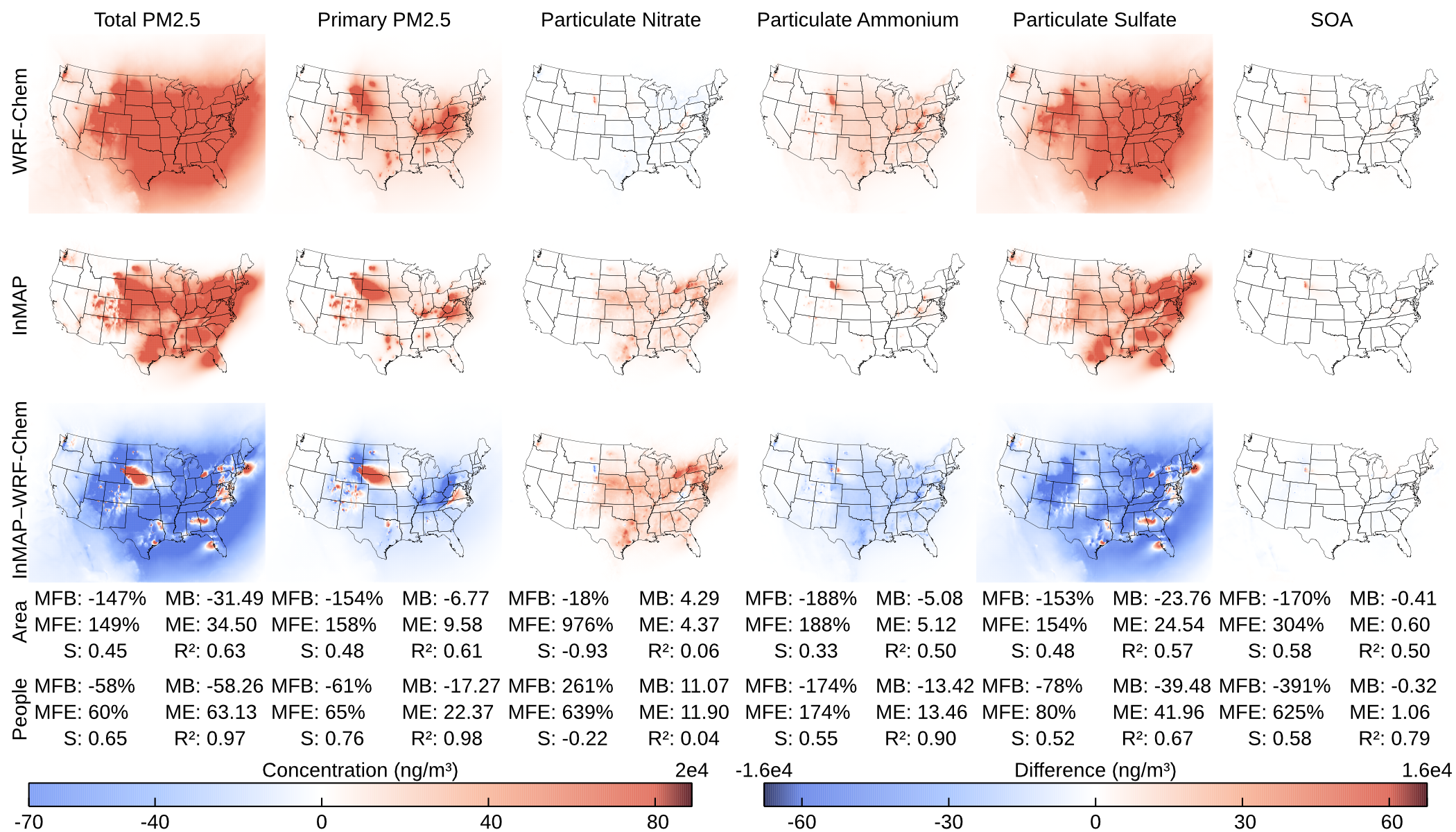

Supplement: S8 Fig — Colors in the first two rows correspond to the legend on the left and colors in the third row correspond to the legend on the right. For ease of viewing, there is a discontinuity at the 99th percentile of concentration values in each color scale. Abbrevations: MFB = mean fractional bias; MFE = mean fractional error; MB = mean bias; ME = mean error; S = slope of regression line; R2 = squared Pearson correlation coefficient. (TIF) [file pone.0176131.s008.tif]

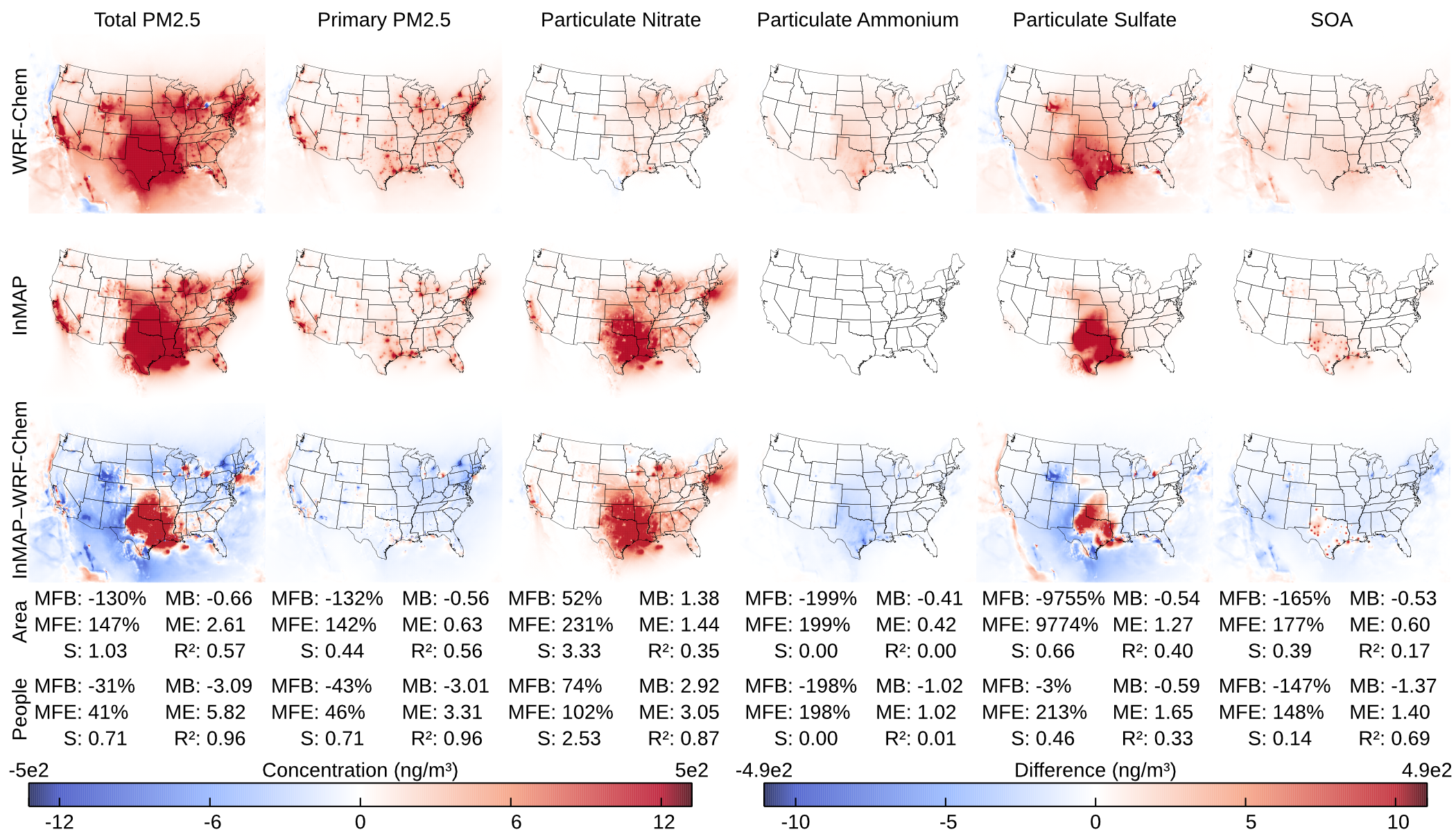

Supplement: S9 Fig — Colors in the first two rows correspond to the legend on the left and colors in the third row correspond to the legend on the right. For ease of viewing, there is a discontinuity at the 99th percentile of concentration values in each color scale. Abbrevations: MFB = mean fractional bias; MFE = mean fractional error; MB = mean bias; ME = mean error; S = slope of regression line; R2 = squared Pearson correlation coefficient. (TIF) [file pone.0176131.s009.tif]

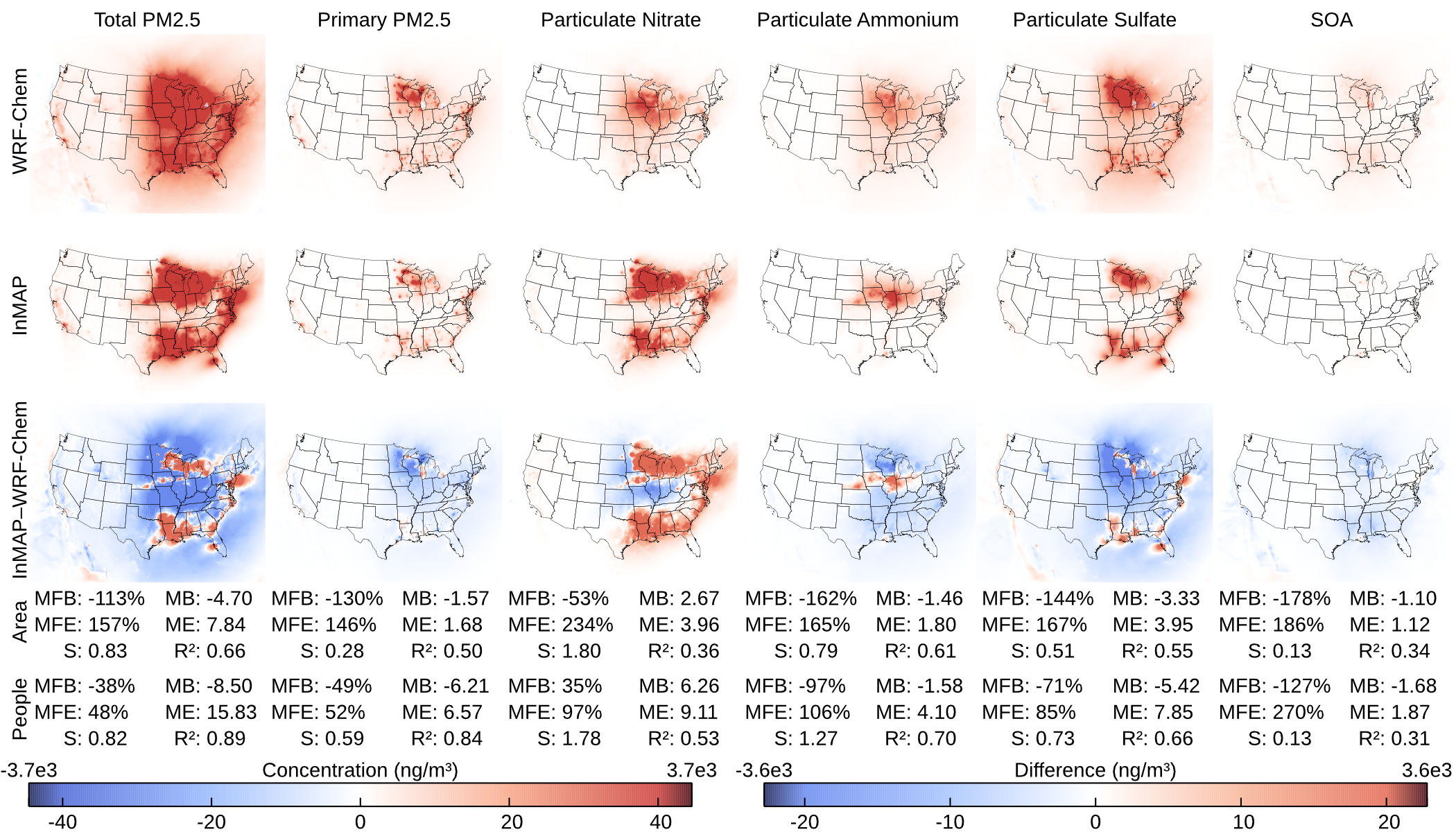

Supplement: S10 Fig — Colors in the first two rows correspond to the legend on the left and colors in the third row correspond to the legend on the right. For ease of viewing, there is a discontinuity at the 99th percentile of concentration values in each color scale. Abbrevations: MFB = mean fractional bias; MFE = mean fractional error; MB = mean bias; ME = mean error; S = slope of regression line; R2 = squared Pearson correlation coefficient. (TIF) [file pone.0176131.s010.tif]

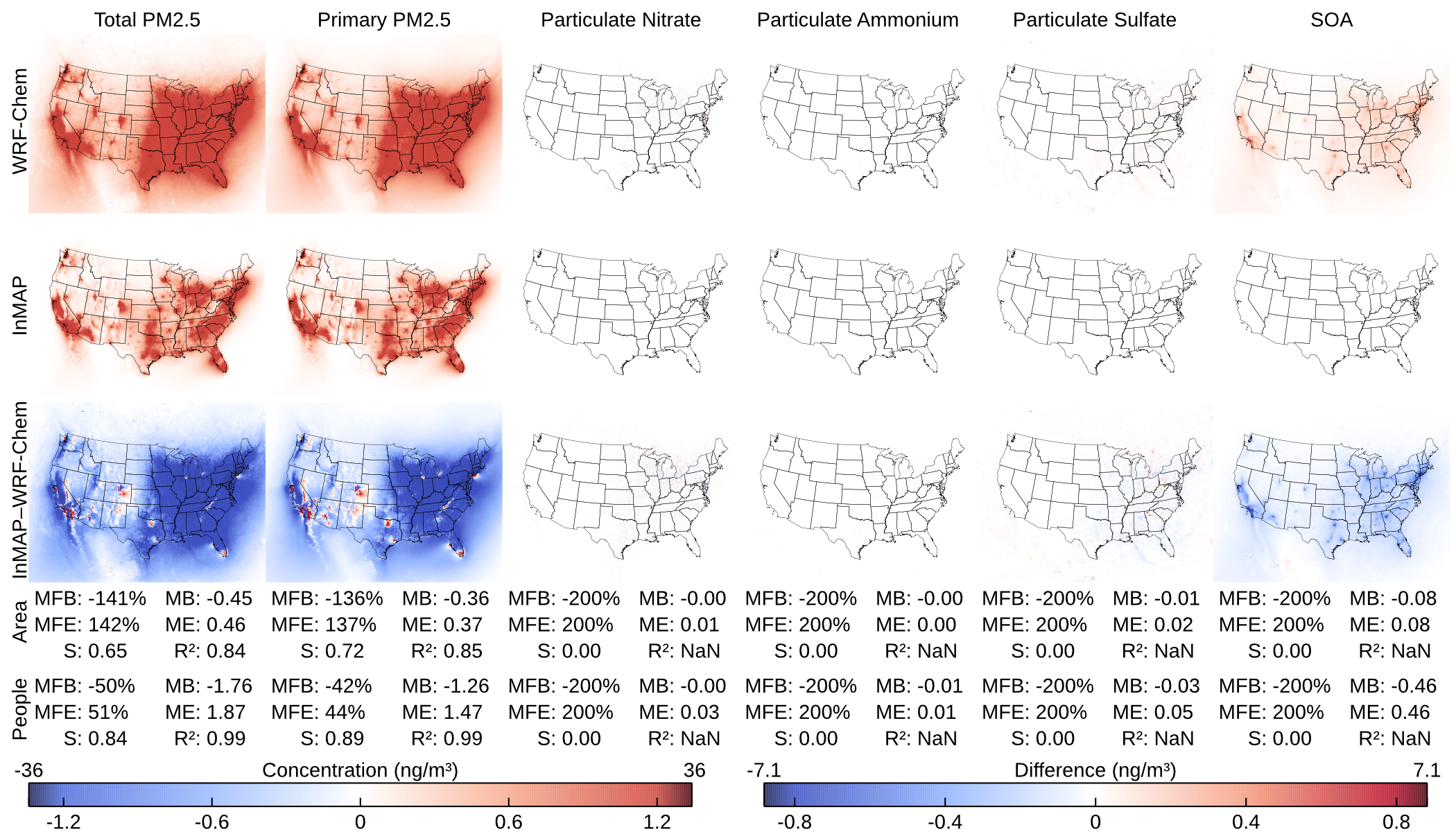

Supplement: S11 Fig — Colors in the first two rows correspond to the legend on the left and colors in the third row correspond to the legend on the right. For ease of viewing, there is a discontinuity at the 99th percentile of concentration values in each color scale. Abbrevations: MFB = mean fractional bias; MFE = mean fractional error; MB = mean bias; ME = mean error; S = slope of regression line; R2 = squared Pearson correlation coefficient. (TIF) [file pone.0176131.s011.tif]

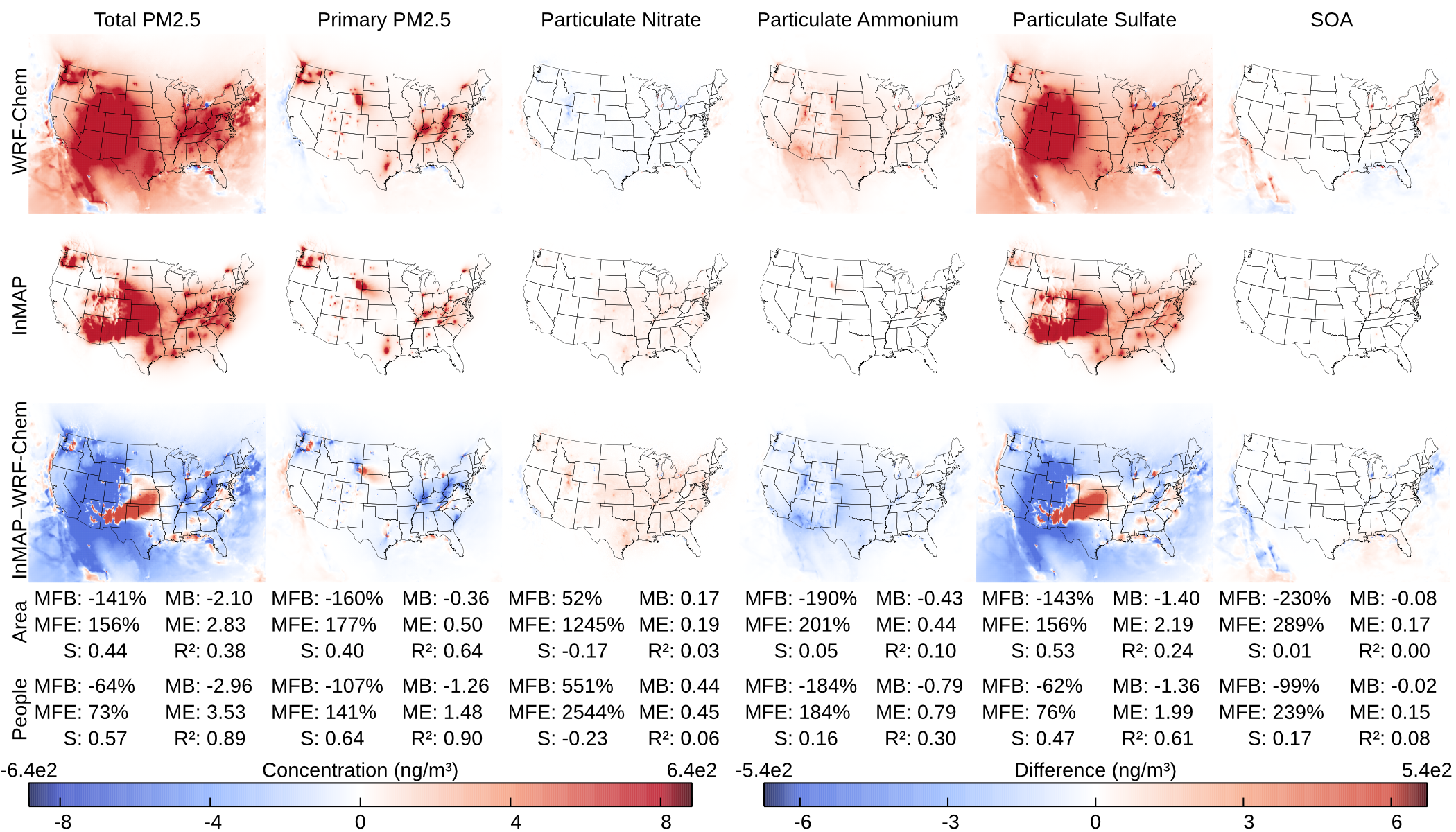

Supplement: S12 Fig — Colors in the first two rows correspond to the legend on the left and colors in the third row correspond to the legend on the right. For ease of viewing, there is a discontinuity at the 99th percentile of concentration values in each color scale. Abbrevations: MFB = mean fractional bias; MFE = mean fractional error; MB = mean bias; ME = mean error; S = slope of regression line; R2 = squared Pearson correlation coefficient. (TIF) [file pone.0176131.s012.tif]

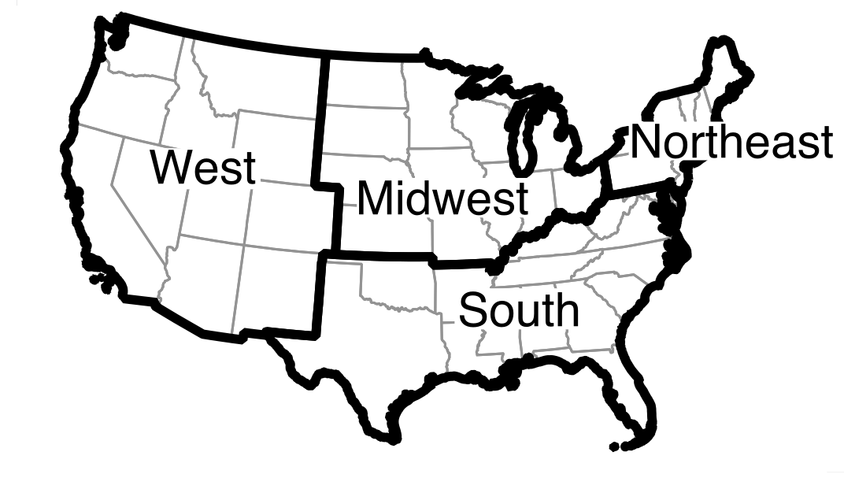

Supplement: S13 Fig — (TIF) [file pone.0176131.s013.tif]

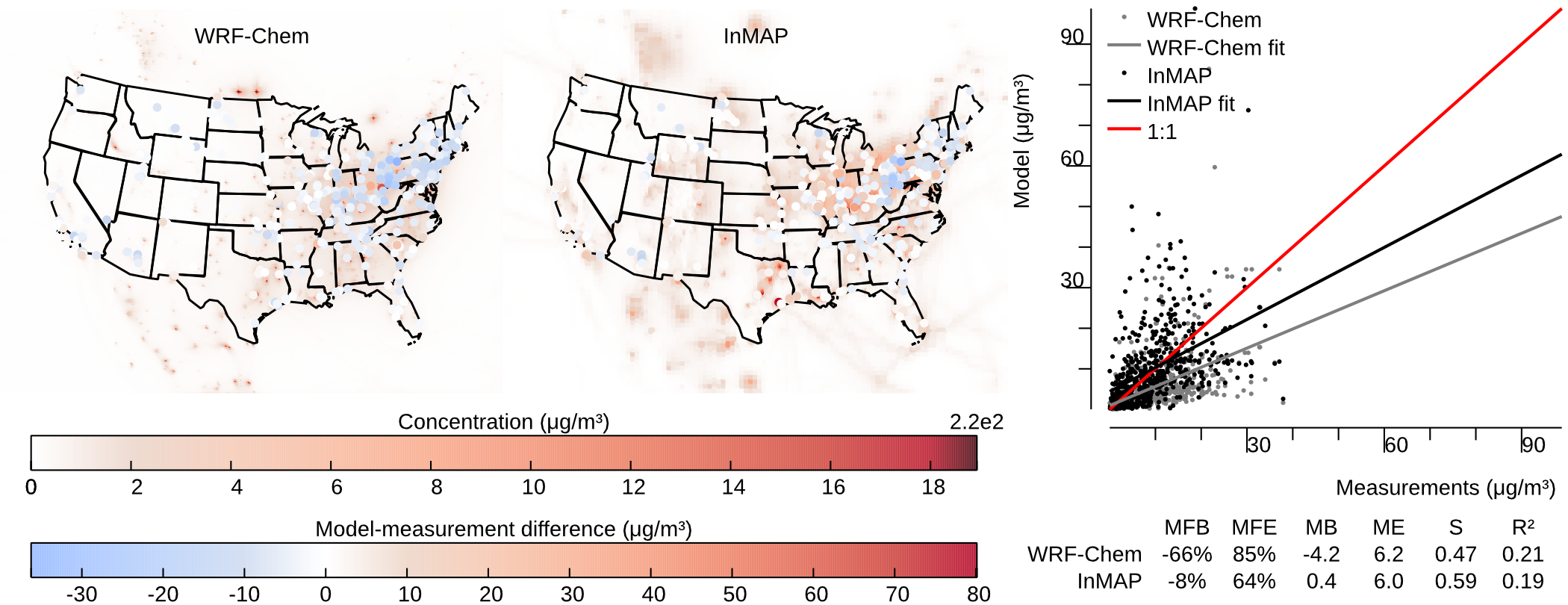

Supplement: S14 Fig — The background colors in the maps represent predicted concentrations, and the colors of the circles on the maps represent the difference between modeled and measured values at measurement locations. Abbrevations: MFB = mean fractional bias; MFE = mean fractional error; MB = mean bias; ME = mean error; MR = model ratio; S = slope of regression line; R2 = squared Pearson correlation coefficient. (TIF) [file pone.0176131.s014.tif]

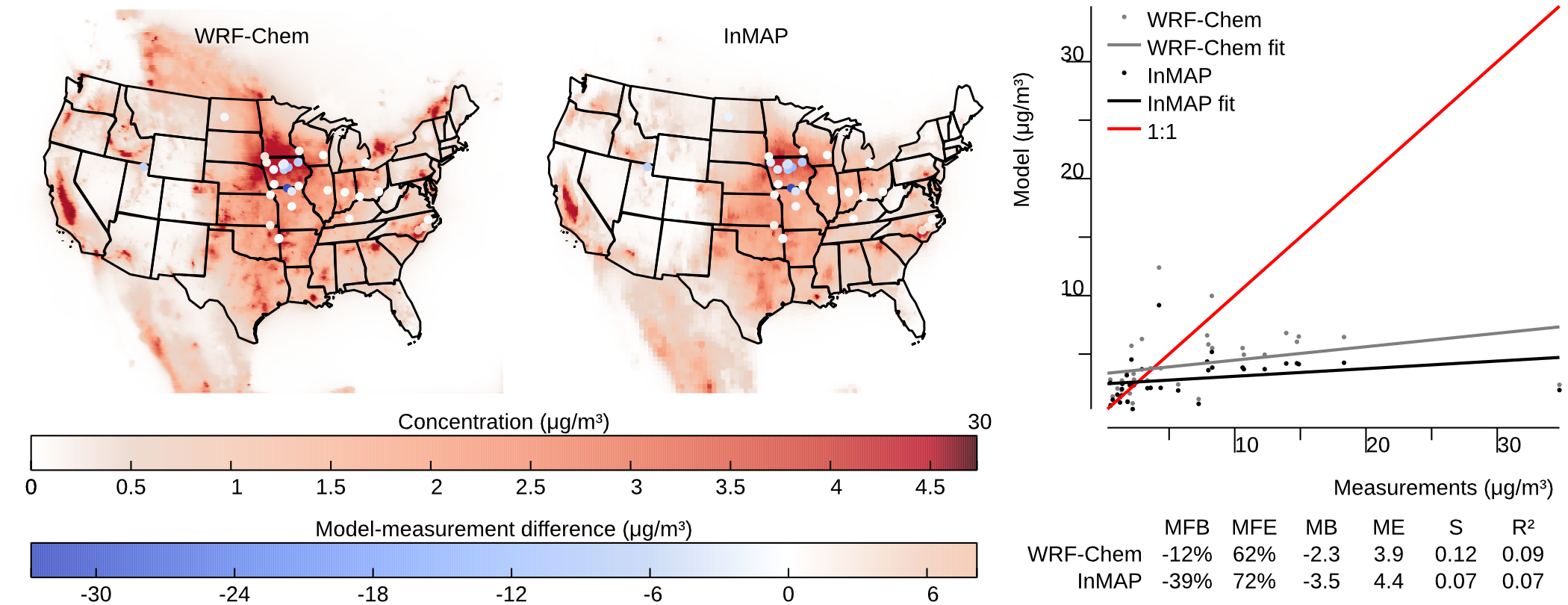

Supplement: S15 Fig — The background colors in the maps represent modeled concentrations, and the colors of the circles on the maps represent the difference between modeled and measured values at measurement locations. Abbrevations: MFB = mean fractional bias; MFE = mean fractional error; MB = mean bias; ME = mean error; MR = model ratio; S = slope of regression line; R2 = squared Pearson correlation coefficient. (TIF) [file pone.0176131.s015.tif]

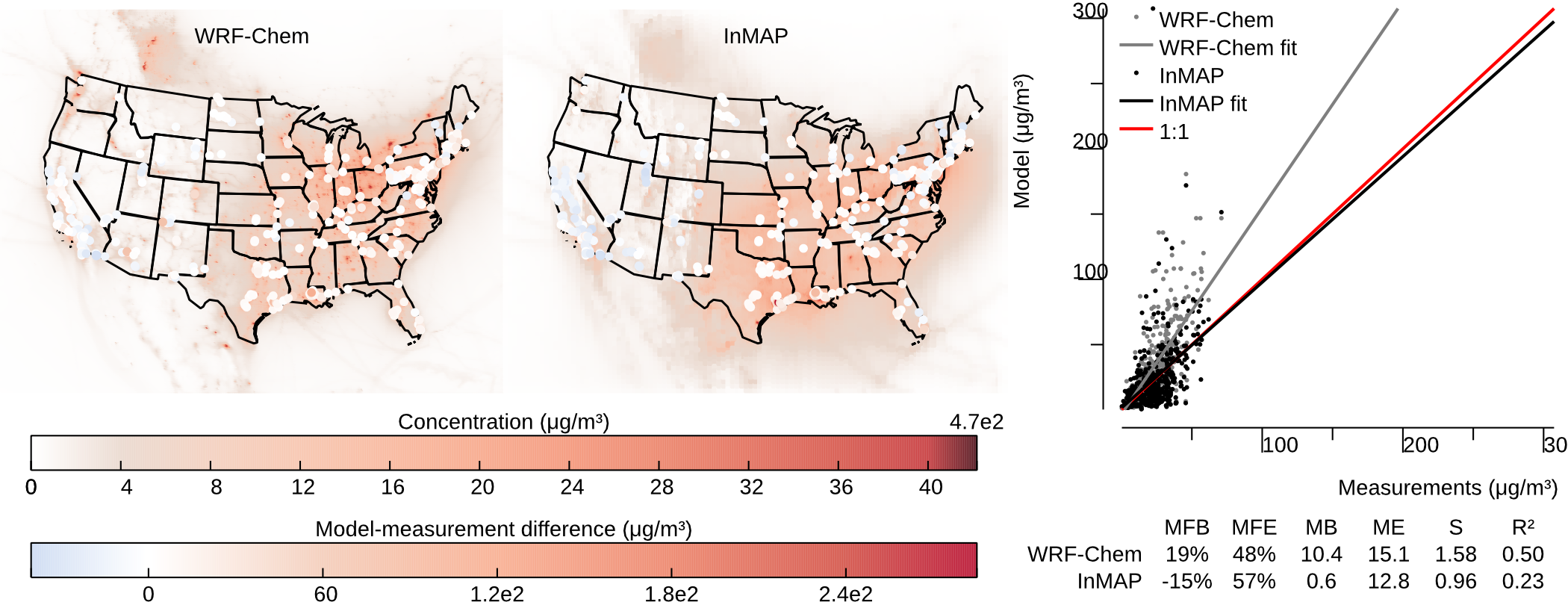

Supplement: S16 Fig — The background colors in the maps represent modeled concentrations, and the colors of the circles on the maps represent the difference between modeled and measured values at measurement locations. Abbrevations: MFB = mean fractional bias; MFE = mean fractional error; MB = mean bias; ME = mean error; MR = model ratio; S = slope of regression line; R2 = squared Pearson correlation coefficient. (TIF) [file pone.0176131.s016.tif]
