# Supplementary material for: InMAP: A model for air pollution interventions
Source: PLoS One. 2017 Apr 19;12(4):e0176131. doi: 10.1371/journal.pone.0176131 (PMC5397056; doi:10.1371/journal.pone.0176131)
Supplement: S1 Appendix — (PDF) [file pone.0176131.s017.pdf]

InMAP: a model for air pollution interventions: supporting information appendix 1

Christopher W. Tessum *[1], Jason D. Hill †[2], and Julian D. Marshall ‡[1]

[1]University of Washington, Seattle, Washington, USA
[2]University of Minnesota, Minneapolis–St. Paul, Minnesota, USA

1 Existing reduced-complexity air quality models

1.1 CTM-based sensitivity models

Several CTM-based tools can reduce the computational requirements of determining how changes in emissions would impact air pollution concentrations. The direct decoupled method (DDM, Zhang et al., 2012; Foley et al., 2014), can, for example, calculate spatially explicit changes in health impacts attributable to changes in overall emissions. The adjoint method (Hakami et al., 2007; Dedoussi and Barrett, 2014), can, for example, calculate how spatially explicit changes in emissions cause changes in overall health impacts. Source apportionment attributes pollutant concentrations or concentration sensitivities among different sources. One example of a source apportionment tool is the Particle Source Apportionment Tool (PSAT) (Wagstrom et al., 2008). All three of these approaches can be computationally inexpensive to use once the original sensitivities are calculated and are likely more accurate than the approach we present here. However, the calculated sensitivities are often not widely adaptable to different use-cases. For instance, changing the spatial distribution of emissions in the case of DDM, the spatial distribution of the human population in the case of the adjoint method, or the sources of interest in the case of source apportionment would require re-running the CTM to create a new set of sensitivities. For this reason, these methods generally are not amenable to use by non-experts.

There additionally exist statistical models based on the results of many CTM runs (e.g., the Response Surface Model, US EPA, 2006; Foley et al., 2014; models based on neural networks or neuro-fuzzy systems, Carnevale et al., 2009; EASIUR, Heo et al., 2016; or the model by Buonocore et al. (2014)); the requirement of many CTM runs renders these models computationally expensive to create and update.

1.2 Gaussian

Gaussian plume models (e.g., AERMOD, Cimorelli et al., 2005) and models that are derived from them (e.g., COBRA, US EPA, 2012; APEEP, Muller and Mendelsohn, 2006; SIM-air, Guttikunda, 2009; or the model developed for the US EPA National Air Toxics Assessment (NATA), Logue et al., 2011) analytically estimate the downwind impact of individual sources or source groups. These models are computationally inexpensive and useful for predicting near-source impacts but are not recommended for predictions of pollution transport over long distances ($> 50\,\text{km}$, US EPA, 2015). Additionally, Gaussian plume models generally cannot robustly represent nonlinear or spatially variable rates of formation and evaporation of secondary $PM_{2.5}$ (Seinfeld and Pandis, 2006).

*ctessum@uw.edu
†hill0408@uw.edu
‡jdmarsh@uw.edu

1.3 Lagrangian

Lagrangian models such as CALPUFF (Scire et al., 2000) and HYSPLIT (Draxler and Hess, 1997) track long range transport from individual sources by tracking a packet of air as it interacts with its surroundings. These models typically are less computationally intensive than CTMs if the number of sources is small, but simulating many individual sources over a broad area can be computationally prohibitive.

1.4 Chemical mass balance

Chemical mass balance models (e.g., CMB: US EPA, 2004) estimate the contribution of different emissions source types to ambient pollution concentrations by analyzing the relative contributions of different chemical tracers and matching them to tracer profiles of known sources. This method is useful for estimating the contribution specific source types, but requires detailed location-specific measurements and can only track contributions from sources with known tracer profiles. Additionally, chemical mass balance models cannot directly predict how changes in emissions would impact concentrations.

References

Buonocore, J. J., Dong, X., Spengler, J. D., Fu, J. S., Levy, J. I. (2014) Using the Community Multiscale Air Quality (CMAQ) model to estimate public health impacts of PM2.5 from individual power plants. Environ. Int., 68, 200–208. doi:10.1016/j.envint.2014.03.031

Carnevale, C., Finzi, G., Pisoni, E., Volta, M. (2009) Neuro-fuzzy and neural network systems for air quality control. Atmos. Env., 43, 4811–4821. doi:10.1016/j.atmosenv.2008.07.064

Cimorelli, A. J., Perry, S. G., Venkatram, A., Weil, J. C., Paine, R. J., Wilson, R. B., Lee, R. F., Peters, W. D., Brode, R. W. (2005) AERMOD: a dispersion model for industrial source applications. Part I: General model formulation and boundary layer characterization. J. Appl. Meteorol., 44, 682–693. doi:10.1175/JAM2227.1

Dedoussi, I. C., Barrett, S. R. H. (2014) Air pollution and early deaths in the United States. Part II: Attribution of PM2.5 exposure to emissions species, time, location and sector. Atmos. Environ., 99, 610–617. doi:10.1016/j.atmosenv.2014.10.033

Draxler, R. R., Hess, G. D. (1997) Description of the HYSPLIT_4 Modeling System. Tech. rep. NOAA Technical Memo. #ERL ARL-224, Silver Spring, MD. `http://www.ciecem.uhu.es/hysplitweb08/document/arl-224.pdf`. Accessed 26 October 2015

Foley, K. M., Napelenok, S. L., Jang, C., Phillips, S., Hubbell, B. J., Fulcher, C. M. (2014) Two reduced form air quality modeling techniques for rapidly calculating pollutant mitigation potential across many sources, locations and precursor emission types. Atmospheric Environment, 98, 283289. doi:10.1016/j.atmosenv.2014.08.046

Guttikunda, S. K. (2009) SIM-air modeling tools. `http://urbanemissions.info/model-tools.html`. Accessed 26 October 2015

Hakami, A., Henze, D. K., Seinfeld, J. H., Singh, K., Sandu, A., Kim, S., Byun, D., Li, Q. (2007) The Adjoint of CMAQ. Environ. Sci. Technol., 41, 7807–7817

Heo J, Adams PJ, Gao HO. Reduced-form modeling of public health impacts of inorganic PM2.5 and precursor emissions. Atmos Env. 2016;137: 80-89. doi:10.1016/j.atmosenv.2016.04.026

Logue, J. M., Small, M. J., Robinson, A. L. (2011) Evaluating the national air toxics assessment (NATA): comparison of predicted and measured air toxics concentrations, risks, and sources in Pittsburgh, Pennsylvania. Atmos. Environ., 45, 476–484. doi:10.1016/j.atmosenv.2010.09.053

Muller, N. Z., Mendelsohn, R. (2006) The Air Pollution Emission Experiments and Policy Analysis Model (APEEP): Technical Appendix. Tech. rep. https://sites.google.com/site/nickmullershomepage/home/ap2-apeep-model-2. Accessed 26 October 2015

Scire, J. S., Strimaitis, D. G., Yamartino, R. J. (2000) A User's Guide for the CALPUFF Dispersion Model. Tech. Rep. Earth Tech, Inc., Concord, MA. http://www.src.com/calpuff/download/CALPUFF_UsersGuide.pdf. Accessed 26 October 2015

Seinfeld, J. H., Pandis, S. N. (2006) Atmospheric Chemistry and Physics: From Air Pollution to Climate Change. Wiley, Hoboken, N. J. 2nd edn.

US EPA (2004) EPA-CMB8.2 Users Manual. Tech. rep. US Environmental Protection Agency #EPA-452/R-04-011. http://www3.epa.gov/ttn/scram/models/receptor/EPA-CMB82Manual.pdf. Accessed 27 January 2016

US EPA (2006) Technical Support Document for the Proposed PM NAAQS Rule: Response Surface Modeling, Tech. Rep. US Environmental Protection Agency, Research Triangle Park, NC. http://www.epa.gov/scram001/reports/pmnaaqs_tsd_rsm_all_021606.pdf. Accessed 26 October 2015

US EPA (2012) User's Manual for the Co-Benefits Risk Assessment (COBRA) Screening Model. Tech. rep. US Environmental Protection Agency, Washington, D.C. http://epa.gov/statelocalclimate/documents/pdf/cobra-2.61-user-manual-july-2013.pdf. Accessed 26 October 2015

US EPA (2015) Revision to the Guideline on Air Quality Models: Enhancements to the AERMOD Dispersion Modeling System and Incorporation of Approaches to Address Ozone and Fine Particulate Matter. Tech. rep. US Environmental Protection Agency #2060-AS54. http://www.epa.gov/ttn/scram/11thmodconf/9930-11-OAR_AppendixW_Proposal.pdf. Accessed 26 October 2015

Wagstrom, K. M., Pandis, S. N., Yarwood, G., Wilson, G. M., Morris, R. E. (2008) Development and application of a computationally efficient particulate matter apportionment algorithm in a three-dimensional chemical transport model. Atmos. Environ., 42, 5650–5659. doi:10.1016/j.atmosenv.2008.03.012

Zhang, W., Capps, S. L., Hu, Y., Nenes, A., Napelenok, S. L., Russell, A. G. (2012) Development of the high-order decoupled direct method in three dimensions for particulate matter: enabling advanced sensitivity analysis in air quality models. Geosci. Model Dev., 5, 355–368. doi:10.5194/gmd-5-355-2012
