# Supplementary material for: InMAP: A model for air pollution interventions
Source: PLoS One. 2017 Apr 19;12(4):e0176131. doi: 10.1371/journal.pone.0176131 (PMC5397056; doi:10.1371/journal.pone.0176131)
Supplement: S2 Appendix — (PDF) [file pone.0176131.s018.pdf]

# InMAP: a model for air pollution interventions: supporting information appendix 2

Christopher W. Tessum *[1], Jason D. Hill †[2], and Julian D. Marshall ‡[1]

[1]University of Washington, Seattle, Washington, USA
[2]University of Minnesota, Minneapolis–St. Paul, Minnesota, USA

## 1 Spatial discretization algorithm

To use numerical integration to solve the chemical and physical equations that describe the processes relevant to air pollution, a model must break up the spatial and temporal domains of interest into finite elements. InMAP spatially discretizes the model domain using a variable resolution rectangular grid, where individual grid cells can nest and telescope between lower and higher resolution based on human population density or other attributes. Grid cell resolution is determined by one of the two algorithms described here. In both algorithms, given a list of possible grid cell sizes, the model domain is first filled with the lowest resolution grid cells (48 km). We will refer to grids produced by the first algorithm as "static variable resolution", as grid cell size does not change during the simulation, and we will refer to grids produced by the second algorithm as "dynamic variable resolution", as grid cell size does change during the simulation.

In the first algorithm, the program next iterates through the grid cells, determining if the population in each grid cell is above a certain threshold level. If the population in the grid cell is above the threshold level, the grid cell is split into grid cells of the next smallest size. The algorithm recurses through this process until either all of the cells are below the population threshold or the smallest specified grid cell size has been reached. The algorithm also has a second constraint, where any of the smallest-size grid cells having a maximum population density greater than a certain threshold level are kept at the finest resolution. This constraint is important where high population density areas are directly adjacent to low population density areas, such as in coastal cities. Because variability in pollutant concentrations decreases with increased height above the ground, all grid cells above a given height cutoff are kept at the lowest model resolution. As shown in Fig. 1 of the main manuscript, we use here a spatial domain which covers the contiguous US, southern Canada, and northern Mexico, with grid cell edge lengths of 48, 24, 12, 4, 2, and 1 km, a population threshold of 40 000 people per grid cell, a population density threshold of 5500 people km$^{-1}$, and a height cutoff of the eighth model layer (approximately 1500 m, chosen because this height is usually above the planetary boundary layer). These settings are chosen to achieve a balance between the spatial detail and model runtime. Other spatial domains are possible: the spatial extent of the modeling domain is only limited by the availability of meteorological and chemical input data. Meteorological and chemical properties in InMAP cells that do not exactly coincide with grid cells in the input data set are taken as the average of all input grid cells that they overlap with.

The second algorithm runs occasionally during the simulation to create a dynamically varying grid rather than before the simulation starts as the first algorithm does. When the second algorithm runs, it first calculates the total human population $P_t$ and the total mass of all pollutants $M_t$ in the simulation domain.

*ctessum@uw.edu
†hill0408@uw.edu
‡jdmarsh@uw.edu

Then for each grid cell, it iterates through each neighboring grid cell, calculating the difference between the population in the grid cell and the population in the neighbor $\Delta P$. Then it iterates through each pollutant species, calculating the sum of concentration differences $\Delta C_t$ as in Eq 1:

$$\Delta C_t = \sum_{i=1}^{n} \left( |C_i - C_{n,i}| \right) \tag{1}$$

where $C$ is concentration, $i$ is one of $n$ pollutant species, and $C_n$ is the concentration in the neighboring grid cell. Then, whether the grid cell should be split into smaller grid cells is decided by the criteria in Eq 2:

$$\Delta C_t \left( V + V_n \right) \Delta P \left( M_t P_t \right)^{-1} > t \tag{2}$$

where $V$ and $V_n$ are the volume of the grid cell and the neighbor, respectively, and $t$ is a threshold value. If Eq 2 is true for the relationship between the grid cell and any of its neighbors, then the grid cell is split into smaller grid cells.

As the model simulation runs, it first starts out with $t = 1$ to ensure no grid cell division and runs until the simulation converges. Then, it sets $t$ to a user-specified value ($t = 1 \times 10^{-11}$ in simulations performed here) and continues to run the simulation until it converges again. This reduces overall model runtime compared with initially setting $t$ to the user-specified value.

# 2 Model computational requirements

Table 1 shows the time required to run InMAP simulations with different emissions scenarios and different grid types and parameters using the computer hardware described in Table 2. Simulation results presented in the main text use a dynamic variable resolution grid with a threshold of 1e-11, which for the National Emissions Inventory scenario has a runtime of approximately two hours.

This computational intensity can be compared to the computational intensity of the WRF-Chem simulation that we used to create preprocessed InMAP inputs, as both the InMAP and WRF-Chem simulations cover the same spatial domain and time period. That simulation was split into eight segments, each of which ran approximately 20 hours on 96 nodes on a high-peformance computing (HPC) cluster, with each node consisting of two quad-core 2.8 GHz Intel Xeon X5560 "Nehalem EP" processors sharing 24 gigabytes of memory. This adds up to approximately 122,880 core-hours for the WRF-Chem simulation, compared to 8 core-hours for an InMAP simulation (2 hours on a 4-core computer), for a speedup factor of approximately 15,000. The hardware used for the WRF-Chem simulations is not identical to the hardware used for the InMAP simulations (shown in Table 2), but it is of similar vintage.

Table 1: InMAP Simulation Run Time for NEI Emissions with a Static and Dynamic Variable Resolution Spatial Grids

| Grid type | Threshold | Cell size[b] | Number of Grid cells | Elapsed time (Hours) |
|---|---|---|---|---|
| static | 4e4; 5.5e3[a] | 11 | 6.0E+05 | 10.1 |
| dynamic | 1e-06 | 48 | 9.3E+03 | 0.4 |
| dynamic | 1e-08 | 36 | 1.0E+04 | 0.6 |
| dynamic | 1e-10 | 12 | 3.2E+04 | 0.8 |
| dynamic | 1e-11 | 7 | 8.0E+04 | 1.9 |
| dynamic | 1e-12 | 4 | 2.0E+05 | 8.1 |

[a] Static variable grid simulations require two parameters for population and population density, respectively.

[b] Cell size is the population-weighted average grid cell horizontal edge length.

Table 2: Computational Hardware Used in Simulations Described in Table 1

| Component | Specification |
|---|---|
| Processor | Intel Core I7-6700 3.40 GHz 8 M Processor Cache 4 LGA 1151 |
| Memory | Kingston HyperX FURY Black 32GB (4x8GB) 2133MHz DDR4 NonECC |
| Motherboard | MSI Intel Skylake H170 LGA 1151 DDR4 USB 3.1 Micro ATX |
| Hard drive | Samsung 850 EVO 1 TB 2.5Inch SATA III Internal SSD |
