# Supplementary material for: InMAP: A model for air pollution interventions
Source: PLoS One. 2017 Apr 19;12(4):e0176131. doi: 10.1371/journal.pone.0176131 (PMC5397056; doi:10.1371/journal.pone.0176131)
Supplement: S1 Table — (PDF) [file pone.0176131.s019.pdf]

InMAP: a model for air pollution interventions: supporting information table 1

Christopher W. Tessum *[1], Jason D. Hill †[2], and Julian D. Marshall ‡[1]

[1]University of Washington, Seattle, Washington, USA
[2]University of Minnesota, Minneapolis–St. Paul, Minnesota, USA

Table 1: Names of WRF-Chem Variables used by the InMAP Preprocessor and their Descriptions

Name(s)	Description and use in InMAP preprocessor
hc5, hc8, olt, oli, tol, xyl, csl, cvasoa1, cvasoa2, cvasoa3, cvasoa4	Anthropogenic VOCs that are SOA precursors; used to determine VOA/SOA partitioning
asoa1i, asoa1j, asoa2i, asoa2j, asoa3i, asoa3j, asoa4i, asoa4j	Anthropogenic SOA; used to determine VOA/SOA partitioning
iso, api, sesq, lim, cvbsoa1, cvbsoa2, cvbsoa3, cvbsoa4	Biogenic VOCs that are SOA precursors; used for model evaluation
bsoa1i, bsoa1j, bsoa2i, bsoa2j, bsoa3i, bsoa3j, bsoa4i, bsoa4j	Biogenic SOA; used for model evaluation
no, no2	Components of NO_x; used to determine NO_x/pNO_3 partitioning
no3ai, no3aj	Components of pNO_3; used to determine NO_x/pNO_3 partitioning
so2, sulf	Gaseous SO_2 and sulfate; used to determine SO_x/pSO_4 partitioning
so4ai, so4aj	Particulate SO_4; used to determine SO_x/pSO_4 partitioning
nh3	Ammonia; used to determine NH_3/pNH_4 partitioning
nh4ai, nh4aj	Particulate Ammonium; used to determine NH_3/pNH_4 partitioning
PM2_5_DRY	Total $PM_{2.5}$ concentration in the baseline simulation; used for model evaluation
U, V, W	Wind fields; used to determine advection and mixing coefficients
PBLH	Planetary boundary layer height; used to determine mixing coefficients
PHB, PH	Base state geopotential and perturbation geopotential; used to calculate layer heights
HFX	Surface heat flux; used to determine mixing and dry deposition
UST	Friction velocity; used to determine mixing and dry deposition
T	Temperature; used to calculate chemical reaction rates and plume rise
PB, P	Base state pressure plus perturbation pressure; used to calculate chemical reaction rates and plume rise
ho, h2o2	Hydroxyl radical and hydrogen peroxide concentrations; used to calculate chemical reaction rates
LU_INDEX	Land use type; used to calculate mixing
QRAIN	Mixing ratio of rain; used to calculate wet deposition
CLDFRA	Fraction of grid cell covered by clouds; used to calculate wet deposition
QCLOUD	Cloud mixing ratio; used to calculate aqueous-phase chemical reaction rates
ALT	Inverse air density; used to calculate mixing and to convert between mixing ratio and mass concentration
SWDOWN, GLW	Downward shortwave and longwave radiative flux at ground level; used to calculate dry deposition

*ctessum@uw.edu
†hill0408@uw.edu
‡jdmarsh@uw.edu
